# Recent Advances in Photosensitizers as Multifunctional Theranostic Agents for Imaging-Guided Photodynamic Therapy of Cancer

**DOI:** 10.7150/thno.62479

**Published:** 2021-08-26

**Authors:** Paromita Sarbadhikary, Blassan P. George, Heidi Abrahamse

**Affiliations:** Laser Research Centre, Faculty of Health Sciences, University of Johannesburg, Doornfontein, South Africa

**Keywords:** Cancer diagnosis, Molecular imaging, Photodynamic therapy, Photosensitizers, Theranostics

## Abstract

In recent years tremendous effort has been invested in the field of cancer diagnosis and treatment with an overall goal of improving cancer management, therapeutic outcome, patient survival, and quality of life. Photodynamic Therapy (PDT), which works on the principle of light-induced activation of photosensitizers (PS) leading to Reactive Oxygen Species (ROS) mediated cancer cell killing has received increased attention as a promising alternative to overcome several limitations of conventional cancer therapies. Compared to conventional therapies, PDT offers the advantages of selectivity, minimal invasiveness, localized treatment, and spatio-temporal control which minimizes the overall therapeutic side effects and can be repeated as needed without interfering with other treatments and inducing treatment resistance. Overall PDT efficacy requires proper planning of various parameters like localization and concentration of PS at the tumor site, light dose, oxygen concentration and heterogeneity of the tumor microenvironment, which can be achieved with advanced imaging techniques. Consequently, there has been tremendous interest in the rationale design of PS formulations to exploit their theranostic potential to unleash the imperative contribution of medical imaging in the context of successful PDT outcomes. Further, recent advances in PS formulations as activatable phototheranostic agents have shown promising potential for finely controlled imaging-guided PDT due to their propensity to specifically turning on diagnostic signals simultaneously with photodynamic effects in response to the tumor-specific stimuli. In this review, we have summarized the recent progress in the development of PS-based multifunctional theranostic agents for biomedical applications in multimodal imaging combined with PDT. We also present the role of different imaging modalities; magnetic resonance, optical, nuclear, acoustic, and photoacoustic in improving the pre-and post-PDT effects. We anticipate that the information presented in this review will encourage future development and design of PSs for improved image-guided PDT for cancer treatment.

## Introduction

As per GLOBOCON report about 19.3 million new cases and 10 million cancer deaths have been estimated worldwide in 2020, being the first or second leading cause of death in most countries 1. Moreover, many clinical studies have reported that the present COVID19 pandemic will further increase the burden of cancer-related mortality rate as a severe acute respiratory syndrome coronavirus 2 (SARS-CoV-2) infection increases the susceptibility and severity of disease course of cancer patients 2. It's almost 250 years of first detection of cancer and 60 years since the first approved cancer therapy, still, researchers worldwide are struggling to find a cure and/or improve the quality of life in cancer patients post-treatment while billions of dollars are spent annually on cancer research 3. Even though much progress has been made in oncological research, there are still many issues that must be addressed to improve cancer therapy and diagnosis. A lot of effort has been focused on two main aspects (a) novel and efficacious anticancer strategies which can overcome the severe side effects caused by conventional treatments, (b) innovative imaging approaches for early diagnosis and real-time functional monitoring to plan, evaluate, and monitor the treatment.

The context of this review is the utilization of photosensitizers (PSs) and their functionalized formulations for several conventional and advanced imaging modalities for the assessment of Photodynamic Therapy (PDT) outcomes. To the best of our knowledge, no systematic discussion has been published which summarizes several imaging modalities in combination with PDT or utilizing PSs as an imaging agent. This review highlights the specific applications and importance of imaging in PDT starting from the conventional fluorescence-guided PDT to more advanced X-ray Computed Tomography (CT), Magnetic Resonance Imaging (MRI), Single-Photon Emission Computed Tomography (SPECT), Positron Emission Tomography (PET), Ultrasound (US), and other optical imaging (OI) techniques combined with PDT for improved efficacies. With this background, the use of both optical and non-optical imaging techniques used for diagnostics as well as to assess PDT outcomes will be discussed in detail. Recently, the focus of advancement in PDT has been shifted towards novel strategies like designing and applications of activatable PS (aPS) which are activated under specific tumor microenvironment and/or stimuli. Several reviews have extensively discussed the important concepts, strategies, significant advances and rationale behind the designing of aPS 4-6. This review will not focus on aPSs as a major topic and have included some relevant examples of aPSs which have been particularly utilized as PDT combined imaging agent. Further, this review will discuss only limited aspects of the PDT providing only an overall overview.

### Photodynamic therapy: Overview

**Figure 1:**
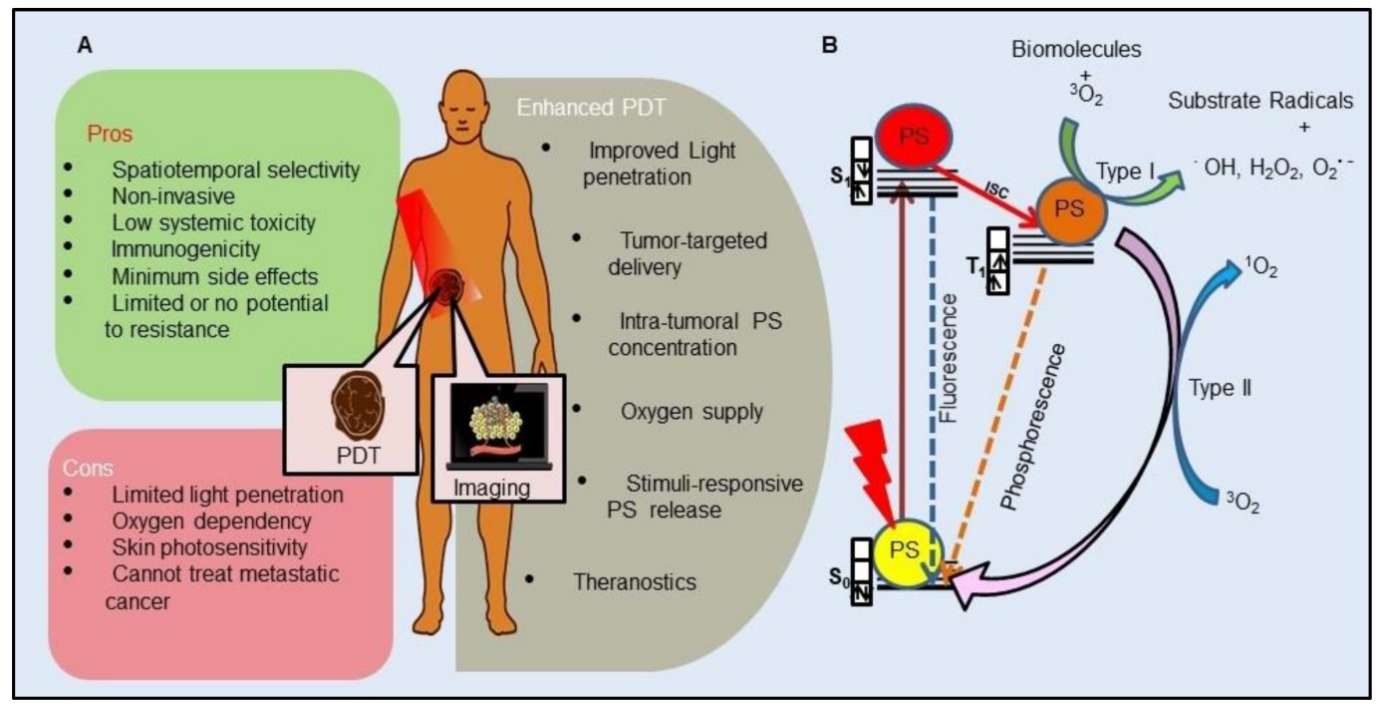
** General schematic representation of Photodynamic Therapy (PDT): A.** Illustration of PDT where photosensitizer (PS) serves as both an imaging agent and a therapeutic agent. Advantages, disadvantages, and different strategies to enhance PDT of cancer. **B.** Modified Jablonski diagram showing the principle of PDT: Absorption of light energy by ground state PS (S_0_) results into its excitation to singlet ^1^PS^*^ (S_1_). Intersystem crossing (ISC) transforms the S_1_ to excited triplet ^3^PS^*^ (T_1_). T_1_ either through electron transfer to cellular biomolecules (Type I) and/or via direct energy transfer to ^3^O_2_ (Type II) results in the production of Reactive Oxygen Species (ROS) to induce cell death.

As an anticancer therapy, PDT is a clinically approved modality and has gained extensive attention owing to its minimal invasiveness, precise controllability, and high spatiotemporal accuracy with minimum side effects (Figure 1A). In principle, PDT is based on the photochemical process where photoactive drugs known as PSs are excited by light of appropriate wavelength (600- 800 nm) to generate cytotoxic reactive oxygen species (ROS) leading to cancer cell death. The wavelength region between 600-800 nm is known as optical or therapeutic window for PDT, which exhibits significantly high optical penetration depth in the tissue, as well as photons of this range have sufficiently high energy to excite the PS. Optical penetration depth of light in tissue is wavelength dependent, which represent distance at which the light intensity reduces to 0.37 of the initial intensity. Endogenous chromophores in tissues like oxyhemoglobin, deoxyhemoglobin, melanin, and water exhibit lowest absorbance in this region, thus having highest penetration depth in tissues. Therefore, due to wavelength-dependent scattering and absorption characteristics of endogenous chromophores, the optical penetration depth of light varies as < 0.5 mm at 400-430 nm, 1 mm at 500 nm, 2-3 mm at 630 nm, and 5-6 mm at 700-800 nm 7.

**Figure 2:**
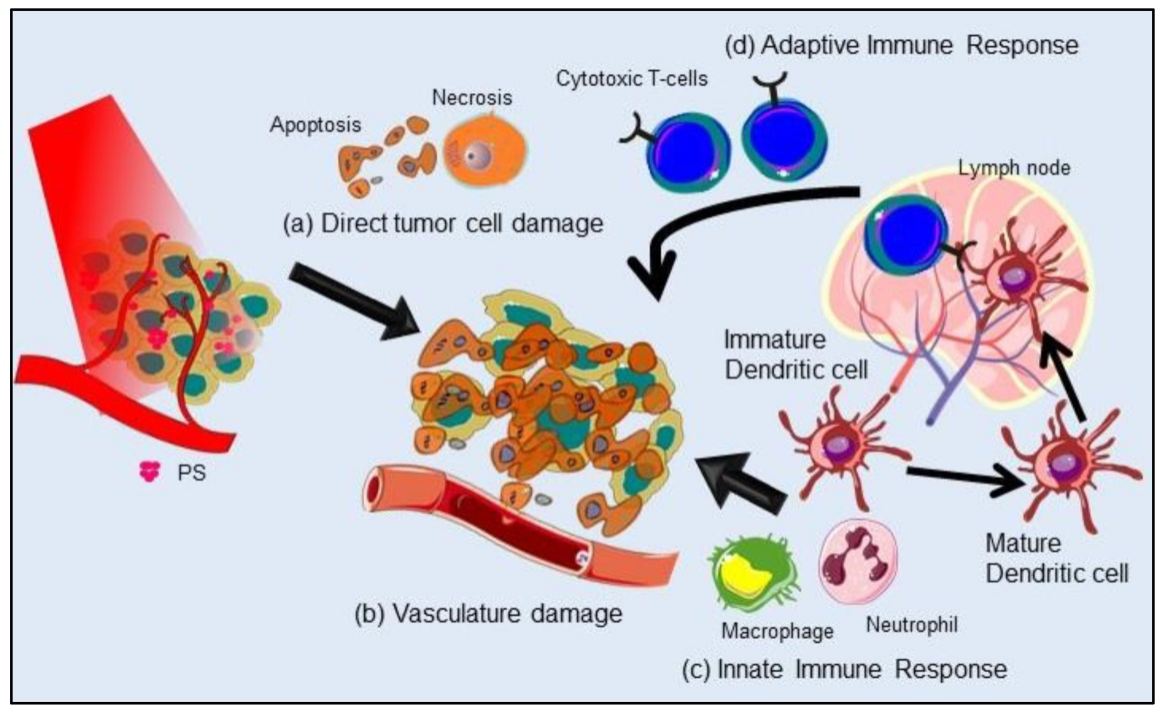
** Photodynamic Therapy (PDT) induced cellular effects and immune responses:** Generation of reactive oxygen species induces (a) direct tumor cell killing predominantly via apoptosis and necrosis, and (b) damages tumor vasculature. In addition, PDT effect is further potentiated by activating both (c) innate and (d) adaptive immune responses against tumor, further eliminating the residual tumor cells. PS: Photosensitizer.

Mechanistically, PDT operates through type I and type II photochemical processes (Figure 1B). By the absorption of light of appropriate energy in the ground state PS (S_0_) transforms to a short-lived excited singlet state (S_1_ or ^1^PS*). Following which either the ^1^PS* returns to its ground state with the emission of longer wavelength light (fluorescence) or can undergo intersystem crossing (ISC) to form a relatively long-lived triplet state (T_1_ or ^3^PS*) by spin inversion of the excited electron. ^3^PS* to S_0_ is spin forbidden transition and thus is a very slow process. ^3^PS* can either relax back to ground state PS, emitting light (phosphorescence) or by transferring energy to other nearby molecules, which forms the basis of type I or type II reactions. The type I process is an oxygen (O_2_)-independent reaction, in which the ^3^PS* directly reacts with the surrounding cellular biomolecules to form free radicals such as hydroxyl radicals (**^.^**OH), superoxide anion radicals (O_2_^-**.**^) and hydrogen peroxides (H_2_O_2_) via electron transfer. While type II is a highly O_2_-dependent process where the ^3^PS* directly transfers its energy to molecular ^3^O_2_ to form highly reactive singlet oxygen (^1^O_2_) 8-10. Generation of ROS causes complex PDT induced tumor response involving direct tumor cell killing, microvascular damage to activation of innate and adaptive immune responses which altogether enhances the therapeutic outcome as represented in Figure 2. PDT induced irreversible oxidative damage in tumor cells leads to cell death predominantly via apoptosis and/or necrosis and to a lesser extent by autophagy. Tumor cell killing effect is further aggravated by vascular damage restricting O_2_ and nutrient supply to tumor mass. Moreover, PDT mediated oxidative stress provokes a strong acute inflammatory reaction at the tumor site due to damaged and leaky tumor vasculature. This includes secretion of proinflammatory cytokines, activation of the complement pathways, and rapid recruitment of neutrophils, dendritic cells and macrophages at the tumor site. Further, phagocytosis of tumor cell debris by phagocytic cells activates the adaptive immune response causing expansion of tumor-sensitized lymphocytes followed by their migration to a damaged tumor site resulting in the elimination of residual tumor cells 10. Furthermore, emerging evidence has shown that PDT induced destruction of tumor stroma and/or microenvironment, increases tumoral drug penetration and can overcome cancer drug resistance and re-sensitize resistant cancer cells to standard therapies 9,11.

Despite its tremendous potential, application of conventional PDT has been restricted as adjuvant therapy and to only superficial tumors due to its inability to treat deep-seated tumors attributed to: poor penetration depth of visible light in tissues, inefficient accumulation of PS inside the tumor mass, tumor hypoxia and heterogeneity. In an effort to establish PDT as a frontline treatment modality and to enhance the therapeutic effect of PDT, innovative approaches are being exploited to circumvent the limitations in the currently used PDT model in clinics. The latest advancements of PDT for cancer therapy have been focused on improving light source to overcome penetration depth issues, stability and targeting ability of PSs, and tumor hypoxia (Figure 1A) 8,12-14.

Although not only PDT, all other conventional and advanced cancer therapeutics have evolved appreciably over the decades and resulted into significant improvements in treatment outcomes and management of cancer patients. However, imaging plays an integral role in clinical cancer care by performing diagnosis, prognosis, screening, staging, treatment planning, monitoring to the assessment of treatment responses in relation to cure and treatment induced toxicity along with improving the basic understanding of the complex cancer biology at molecular to the cellular level. Innovations in single-model and multimodal conventional and novel imaging modalities offer promising and exceptional opportunities towards targeted, precision, and personalized cancer therapy. The following section provides a comprehensive overview of clinically approved imaging techniques in cancer therapy and their unique imaging potency and intrinsic limitations. Further, the utility of these imaging techniques in planning, designing, monitoring, and assessment, for enhancing the effectiveness of PDT outcomes have been briefly discussed in the following section.

### Cancer Imaging Modalities

Other than advancement in targeted anticancer therapies, another field in which tremendous efforts are being investigated is advanced cancer imaging modalities and development of multi-functional theranostic systems for combined cancer diagnosis and therapy. Medical imaging is an indispensable component of clinical cancer treatment paradigm and plays an important role in all phases of cancer management starting from screening, detection, staging, prognosis, therapy planning, guidance and finally to monitoring therapy response, recurrence and palliation. Main requirements of cancer diagnostics include minimal to non-invasiveness, imaging convenience without tissue destruction, real-time monitoring, functioning over wide ranges of time and size, and should furnish morphological, structural, metabolic and functional information from molecular to cellular to organ to organism levels 15,16. Over the years, cancer imaging techniques have made significant progress from conventional methods yielding structural information to more advanced and relatively recent functional and molecular imaging technologies. Traditional imaging methods, such as MRI, CT and US basically provide structural information primarily size, shape, morphology and location of internal body parts. These imaging techniques furnish information about physical or anatomical abnormalities in the structure like bones, organs, and blood vessels without providing any detailed information thus often needed to confirm a diagnosis by invasive methods like biopsy. Moreover, tumors need to attain a certain size to be imagined anatomically which limits the early detection of cancer and cannot be used for real-time monitoring during the therapeutic regime. While functional imaging which is frequently associated with molecular imaging examines the physiology of diverse and dynamic bioprocess at the cellular or organ level like detection of changes in blood circulation, diffusion, perfusion and bio-distribution of drugs. Functional imaging techniques include diffusion MR techniques, perfusion weighted imaging, functional MRI and pharmacological MRI. Molecular imaging is a more advanced and emerging biomedical research field that enables visualization, characterization and quantification of biological processes taking place at the molecular, subcellular and cellular levels within the living system 16-18. Molecular imaging allows repeated *in vivo* detection of various critical molecular features of cancer hallmarks, such as proliferation, metabolism, hypoxia, angiogenesis and apoptosis. More importantly, molecular imaging has a significant role, in personalized cancer treatment, owing to its involvement in early detection and/or real-time monitoring of molecular changes occurring in cancer cells or tissue and consequently, identifying changes in individual patients both at pre- and post-treatment. This further allows for planning and choice of most appropriate therapy, doses etc., depending on the stage and biological features of the tumor 18,19.

Molecular imaging includes US, MRI, CT, OI, nuclear imaging involving PET and SPECT. Other than these techniques, some more advanced imaging modalities like photoacoustic imaging (PAI), Surface-enhanced Raman Scattering (SERS) imaging, upconversion luminescence (UCL) and cerenkov luminescence imaging are gaining interest in cancer management. However, each imaging modality has pros and cons, based on their unique sensitivity, spatial resolution, temporal resolution, penetration depth etc., (Table 1). Thus, no single modal imaging technique is sufficient enough to acquire all the required information and the choice of imaging technique primarily depends on the biological system being studied and the physiological question being asked 16. A multimodality imaging technique combined with two or more imaging methods can overcome the intrinsic limitations of each modality. Furthermore, this approach results in obtaining complementary, effective, high quality, and accurate information about the anatomical, physical, structural and physiological function for cancer diagnosis and treatment. Moreover, clinical implementation of combined PET/MR and PET/CT scanners, have made the implementation of multimodal imaging more feasible 17,20.

### Role of Imaging in Photodynamic Therapy

**Figure 3:**
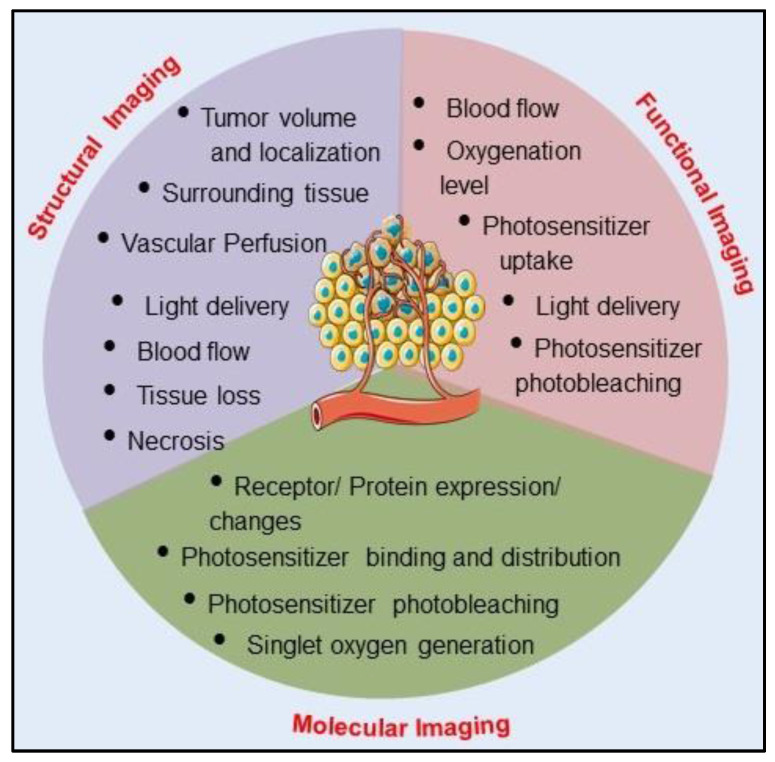
A schematic illustration depicting the roles of structural, functional and molecular imaging in guiding pre-treatment planning, therapy monitoring, and outcome assessment in Photodynamic Therapy.

Like any other therapy, the success of PDT efficacy is also based on the accuracy of pre- and post-PDT dosimetric and monitoring parameters for predicting therapeutic response and planning of subsequent therapeutic schedules. PDT based dosimetry parameters include: (1) pretreatment tumor parameters, such as tumor size, vascular density, oxygen distribution at the tumor site and inter-and intratumoral heterogeneity, (2) real-time therapeutic monitoring parameters including uptake and concentration of PS at the tumor site, its intra and extracellular spatial localization, light dose, and distribution, and (3) post-therapy monitoring of response, recurrence and palliation. Assessment of therapeutic outcome by employing imaging techniques post PDT treatment in tumor tissue provides evidence of necrosis, apoptosis, tumor damage and blood vessel occlusion. Due to the intrinsic fluorescence property of PS, OI based on PS fluorescence imaging had become the standard imaging technique in every stage of PDT, from detection to treatment planning and the outcome in clinical settings. As compared to conventional imaging modalities fluorescence-guided imaging technique offers several advantages of better spatial and temporal imaging resolution along with being a much safer imaging modality. However, fluorescence imaging limits the imaging depth up to few micrometers, thus can provide information of superficial structures only and fails to provide information in the 3D tumor volume. This inherent limitation of fluorescence imaging recommends the importance of other imaging modalities and/or multimodality imaging which can complement the uniqueness of fluorescence imaging by depth and volume resolved imaging approaches 21,22. Pre and post PDT treatment and diagnostics with most commonly used clinical imaging techniques PET, MRI and CT provides significant information related to tumor localization and volume. However, without the use of exogenous contrast agents, these imaging methods suffer from the limitation of lack of resolving power of microcirculatory activity, thus makes it difficult to predict the actual dose required for the treatment outcome. Therefore, as mentioned in the previous section and Table 1, each and every imaging modality has certain strengths and limitations, and the choice for single or combination of imaging techniques in PDT entirely depends on the specific and relevant information required at clinical level. Table 2 represents conventional CT, PET, MRI, and advanced US, PAI, Optical Coherence Tomography (OCT), Fluorescence imaging (FLI) and other imaging techniques that have been successfully used in preclinical and clinical settings for the assessment of various structural and functional information to guide, monitor and evaluate PDT responses 11,21,22. The relationship between structural, functional and molecular imaging approaches and their roles in pretreatment planning, monitoring therapy, and assessment of outcomes are schematically presented in Figure 3. As already mentioned, every imaging modality suffers from their intrinsic drawbacks of resolution, sensitivity and specificity. Thus, multimodal imaging and/or PDT combined imaging has the potential to provide optimism for the future of imaging in PDT and development of personalized patient treatments.

## Theranostics: ''Multifunctional Agents'' for Image-guided Photodynamic Therapy

### Application of Photosensitizers in Photodynamic Therapy and Imaging

**Figure 4:**
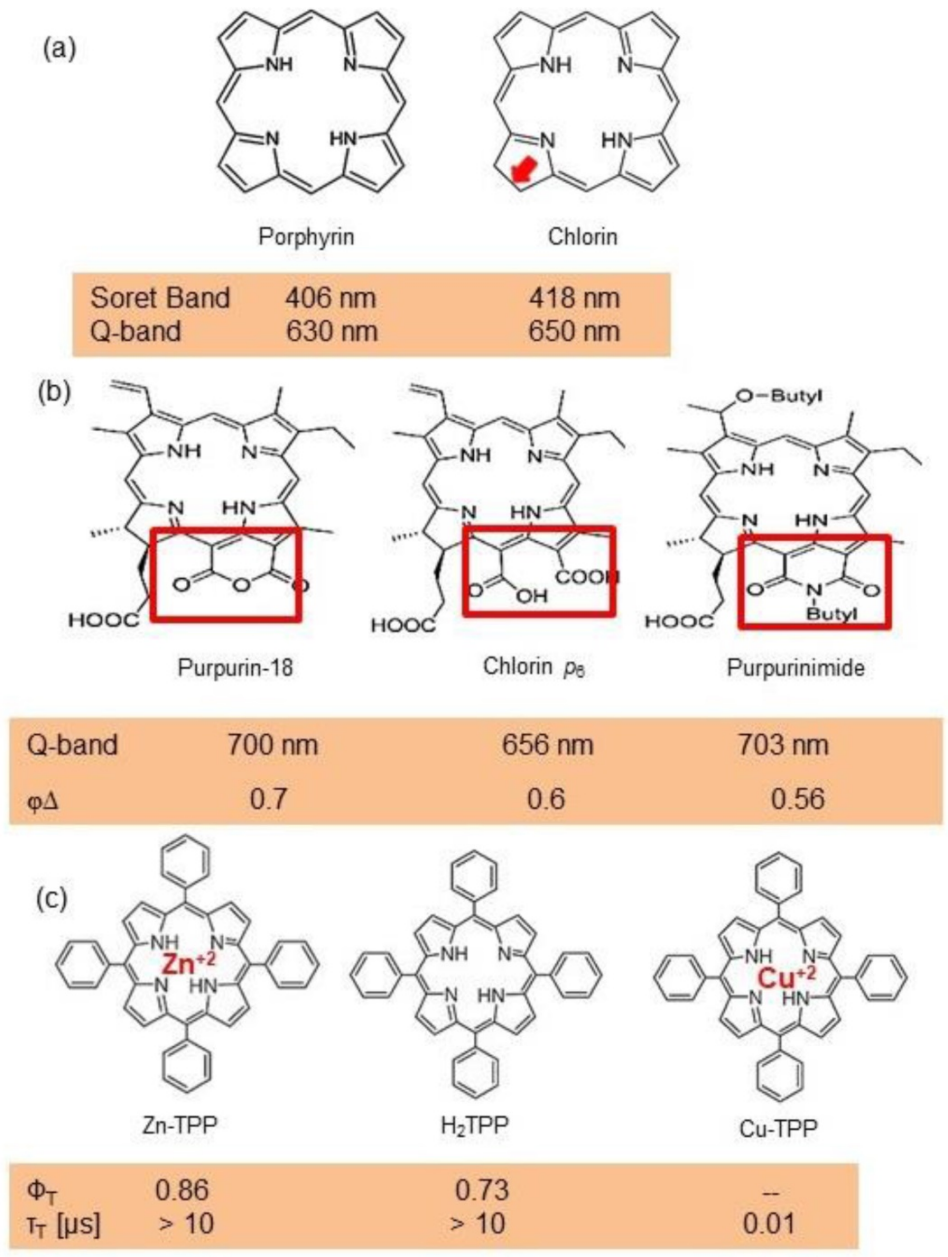
** Chemical modifications of photosensitizer molecules with resulting photophysical and photochemical changes:** (a) reduction of main macrocyclic porphyrin ring results in red shift of Q band of tetrapyrrole photosensitizer, (b) peripheral modification and (c) central metal coordination of tetrapyrrole ring induce changes in singlet oxygen quantum yield (φ∆), triplet quantum yield (Φ_T_) and triplet state lifetime (τ_T_) depending on the type of side groups and central metal (diamagnetic or paramagnetic). *Soret band: The strong absorption band of PS in the blue wavelength region of the visible spectrum due to the S_0_ to S_2_ transition. Q band: The weak absorption band of PS in longer wavelength region i.e red or far red, due to S_0_ to S_1_ transition.φ∆: Quantitative measure of PS efficiency to convert O_2_ into ^1^O_2_ upon photoexcitation. Φ_T_: Number of PS molecules that undergoes singlet to triplet state transition with per photon absorption. PS: Photosensitizer*

In PDT, other than light dose and oxygen concentration, PSs play a critical role in the photochemical reactions to dictate the overall therapeutic outcome of PDT. Ideal PS must possess several ideal photophysical, chemical and pharmacokinetic properties to determine their application and efficiency as PDT agents. Ideally a PS should be in its chemically pure form with high storage stability. Photophysical characteristics of ideal PS should include strong absorption in the optical window region, substantially high triplet state and ROS quantum yield upon irradiation. It should also have preferential uptake by tumor tissue, minimal dark toxicity and rapid clearance from normal tissues to minimize the phototoxic side effects 23. Most of the PSs used in PDT of cancer are based on the tetrapyrrole macrocyclic structure having extended π-electron systems responsible for their unique photophysical and photochemical properties. As illustrated in Figure 4 these photophysical and photochemical properties can be strategically tuned by: (i) chemical modification of the main macrocyclic porphyrin ring, (ii) introducing various functional groups/substituents as peripheral modification and (iii) coordination of metal ions in the centre of tetrapyrrolic ring 24. Although photophysical and photochemical properties of PSs can be easily modified by chemical manipulation, while on the other hand, their pharmacokinetic profiles cannot be easily controlled. As shown in Table 3, numerous PSs are commercially available and have received clinical approval or have entered clinical trials for the treatment of various types of cancer. Despite several natural and synthetic PSs have been reported, not many PS meets all the required ideal properties thus significantly limits their clinical use. The rationale designing of novel PS with desirable properties remains a big challenge. In recent years, several innovative PS designs and strategies are being explored to improve the treatment of deep-seated or thick tumors, stability and targeting ability of PSs, ROS production efficiency, tumor hypoxia, aPSs and nanosystem-based PS formulations for improving PDT efficacy 8,23,25-30.

Furthermore, utilization of PSs are not restricted solely to therapeutic PDT purpose. Being photoactive molecules, many PSs are inherently brightly fluorescent with their fluorescence emission in far red or Near Infrared Radiation (NIR) region of electromagnetic spectra. Hence, PSs holds promise for *in vivo* FLI and is generally employed to monitor tumoral PS uptake and fluorescence-guided surgery (FGS) as is already approved for both bladder and brain cancers in Europe. In addition, FLI also allows follow-up PDT to remove any residual tumor cells that could cause recurrence and evaluating the outcome of treatment. Thus, the fluorescence property of PSs aids in useful dosimetric parameters like determining PS localization and uptake by tumor tissue in real-time and post PDT monitoring 22,27.

With the advancement in medicine, drug designing has revolutionized from the utilization of different drugs for therapy and diagnostics separately to theranostics by integration of therapeutic and diagnostics potential into a single drug molecule 31. In 1998, Funkhouser coined the term “Theranostic” defining those materials that combine both therapeutic approach and diagnostic imaging in a single entity, so that both the agents are delivered at the same time will show the same biodistribution pattern 31,32. The theranostic field has the advantage of tuning the therapy and dose by gaining the ability to image and monitor the diseased tissue, delivery kinetics, and drug efficacy. Thus, due to its real-time monitoring of therapeutic outcome, theranostics have advanced in the biomedical field for the effective and personalized treatment approach 31.

Traditional theranostic agents are basically simple combinations of imaging and therapeutic agents which are always used for both therapeutic effects and imaging signals. The drawbacks of these theranostic agents are its limited signal-to-noise ratio (SNR) and lack of selectivity or specificity in the disease sites. To overcome this issue, an activatable theranostic approach is being researched where an inactive agent could be specifically turned on at the target site by certain stimuli or reactions for simultaneous diagnostics and therapeutic application 33,34. These activatable theranostic agents are endowed with a lower limit of detection, real-time detection of biomarkers, lower toxicity to the normal tissues, higher drug bioavailability and higher SNR 34. In recent years, optical-based phototheranostics have gained increasing attention, due to the advantages of minimal invasiveness, local treatment and spatio-temporal delivery of light reducing the collateral damage to normal surrounding tissues 35.

**Figure 5:**
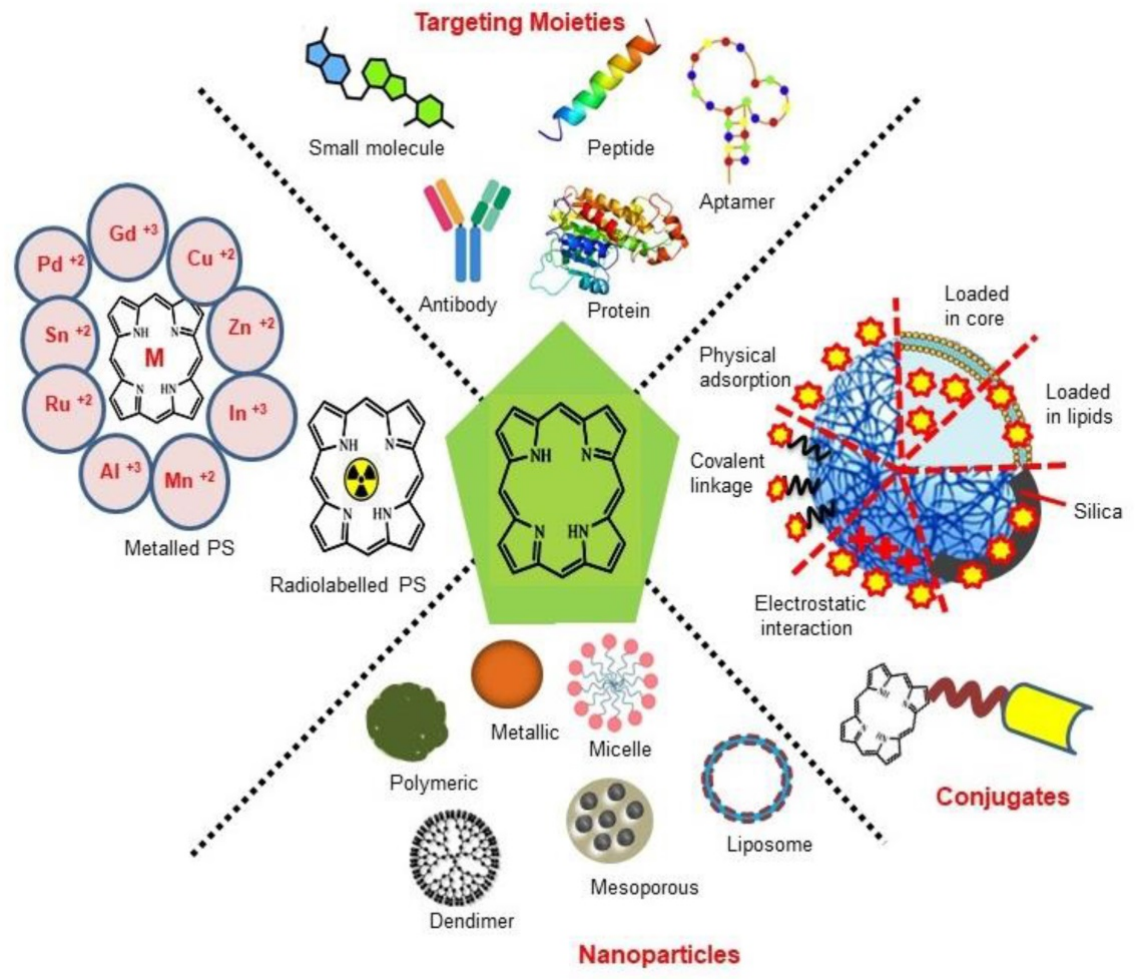
** The structural designing of photosensitizer for therapy and imaging:** Non metallated and metallated (radioactive or nonradioactive isotope) in the form of conjugates, linked with targeting moiety and nanoparticles for application in image-guided Photodynamic Therapy.

Although the concept of phototheranostics is recent, PDT already has proven theranostic applications due to the uniqueness of PSs fluorescence property, thus combining both therapeutic and imaging agent in a single molecule. As already discussed, fluorescence-based imaging in PDT has also shown to have multiple significance including diagnostics, dosimetry, monitoring, treatment assessment, therapy guidance and mechanistic studies 21. FLI can provide cellular-level information with high sensitivity, however its widespread application in imaging field is limited due to poor penetration depth of light in tissues and low resolution. But this has not discouraged the use of PSs as theranostic agents due to their several favorable features like preferential uptake in tumors, relatively low *in vivo* toxicity and more importantly its ease and straightforward functionalization chemistry. Thus, rationale designing with PSs, their derivatives, metallated counterparts and nanoformulations are actively being explored for several other imaging modalities like PET, SPECT, MRI, US, PAI, CT etc. for image-guided PDT (Figure 5) 3,11,36-39. Figure 5 shows some representative designing strategies of PS for theranostics, this includes (1) metalled, non metalled and radiolabelled PS molecules, (2) nanoformulations fabricated using organic, inorganic, lipid and protein compounds, and (3) free or nanoparticle-based PS conjugated with targeting moieties to specifically target the tumor tissues. Targeting moieties including proteins (mainly antibodies and their fragments), peptides, nucleic acids (aptamers) and other small molecules are specifically used to guide PSs to specific tumor tissues. PS conjugates includes conjugating the free PS and/or decorating their nanoformulations with functional and targeting moieties.

### Optical Imaging Combined Photodynamic Therapy

Over the past decades, diagnostic imaging has gained interest and has advanced steadily for the improvement and management of cancer patient care. As an alternative to conventional imaging modalities, OI techniques employing light (usually visible) to extract diagnostic information from light-tissue interactions have emerged as a safe and non-invasive technique for *in vivo* imaging 40. Further, in comparison to the conventional imaging modalities certain interesting features such as ease of detection, high spatial and temporal resolutions, real-time evaluation and availability of a wide variety of light activated contrast agents uniquely make the OI platform an advantageous tool for clinical diagnosis and surgical applications 41. Advancements in technologies have upgraded imaging of tumor lesions due to improved tissue penetration, sensitivity, and specificity by optical methods 41,42. Due to the inherent fluorescence property of PS, their free form or the nanoformulations are widely used for *in vivo* conventional FLI in combination with real-time monitoring of surgery and targeted PDT by observing the PS accumulation. Better insight into the photophysical properties of PSs, their *in vivo* intermolecular interactions and the scope of designing newer formulations has widened their applications from conventional FLI to other advanced OI techniques like SERS imaging, UCL imaging (UCLI) and PAI. In this section, different OI modalities combined with PDT have been discussed separately.

#### Fluorescence Imaging Combined Photodynamic Therapy

FLI has become a highly adoptable clinical imaging modality for tumor detection to image-guided surgery 41. Benefits of FLI involves: (a) high spatial resolution and sensitive investigation of both functional and structural changes, (b) non-invasive, safe technique using non-ionizing radiation, (c) making use of portable and low cost clinical equipments, (d) provide real-time images, (e) can be customized with microscopy and endoscopy to provide both microscopic and macroscopic information and (f) provide quantitative information for aimed diagnosis and follow-up 43. However, the primary limitation of FLI is the poor penetration depth of light in biological tissues due to the scattering and absorption properties of tissue. Scattering results in loss of directionality of light, thus results in a blurred and low resolution images. Further the light absorbing endogenous molecules (melanin and hemoglobin) results in a reduction of light intensity, therefore significantly decreases the SNR in the visible range 44. Thus, due to the drawbacks of autofluorescence signals and light scattering, the applications and designing of effective fluorescent agents remains a challenge. While the SNR can be improved by the use of long-lived fluorescent agents which can eliminate the short-lived background fluorescence interference during the imaging 45. Owing to inherent optical absorption and fluorescence properties of PSs used in PDT, PSs offers their application as theranostic agents. In 1920s, Policard showed the first indication of PDT-related imaging via fluorescence with his observation that Hematoporphyrin (Hp) localization in tumor tissues was more fluorescent than normal ones in a rat sarcoma model. But it was only in the 1950s, Rassmussan-Taxdal and colleagues successfully proved the FLI technique clinically, by injecting Hp to patients prior to the excision of benign and malignant lesions 21.

Combined with its fluorescence property, preferential accumulation of PSs in neoplastic tissues has been shown to be inherently well-suited for selective fluorescence-based visualization of tumors to demarcate the boundaries of cancerous and healthy tissues, photodynamic diagnosis (PDD) and molecular imaging, an approach termed as PS fluorescence detection (PFD). PFD has been mainly applied for real-time imaging for FGS 46. Currently, 3 PSs 5-aminolevulinic acid (5-ALA), methylene blue (MB) and indocyanine green (ICG), have been successfully applied clinically for FGS 47. ALA and its derivative 5-ALA Hexylester (Hexvix®) have already approved for bladder cancer imaging. Photofrin, a derivative of porphyrin has also been used for the detection of various malignant and premalignant tumors. Upon irradiation with a 405 nm full-color endoscopic FLI system, red fluorescence of Photofrin was shown to significantly overcome the green autofluorescence of tumor mass 48. A single centre Phase III randomized controlled trial showed that ALA and Photofrin FGS and repetitive PDT offered a beneficial therapeutic advantage to patients with Glioblastoma multiforme without any risks 49. Another promising randomized controlled multicentre phase III trial evaluated the efficacy of ALA based FGS in enabling intraoperative visualization and resection of malignant glioma thus leading to an overall improvement in progression-free survival 50. In a recent nonrandomized pilot study, a treatment regime including 5-ALA fluorescence-guided maximal resection followed by 5-ALA intraoperative PDT showed to be a good treatment option with prolonged survival in recurrent Glioblastoma multiforme patients 51.

Clinically, standard diagnostic imaging equipment such as endoscopes, laparoscopes, cystoscopes and neurosurgical microscopes are being implemented for FLI. Most of the PSs possess significantly high fluorescence quantum yield, which makes these PSs eligible candidates as dual-functional theranostic agents for clinical applications, however, Tookad is an exception. Although as most free forms of PSs lack sufficient biological stability, solubility and specificity, which has restricted the inclusion of only a few PSs into clinical applications as theranostic agents 21,22,47. Compared to conventional imaging modalities such X-ray CT, MRI and PET, because of nonradioactive properties, fluorescence-based imaging is much safer and causes less damage in patients. However, due to the poor penetration depth of visible and NIR light of few millimeters in tissue, the FLI can only reach a limited depth as compared to nuclear imaging modalities 47. Other than the limitation of in-depth penetration, as compared to the volume sensitive techniques like CT, MRI and PET, FLI is a surface-sensitive technique which cannot provide structural details during the process of resection, thus fails for tumor lesions with considerable subsurface depth 21,46. To address these inherent limitations of PFD, extensive research is being focused on developing novel, more potent and tumor-specific PSs with potential clinical applications. In this regard, some of the approaches on PSs development, which are being explored includes: (1) enhancement of PS fluorescence, (2) novel PS with long excitation wavelength; (3) implementation of multimodal imaging approach based on PFD, complemented with depth-resolved structural imaging modality. The recent progression of multifunctional platforms or delivery systems has also been explored for designing and delivery of PSs combining PFD with PDT. These multifunctional delivery systems also offer the advantage of efficient delivery of the hydrophobic PSs, to avoid reduction in their photodynamic efficacy due to self-aggregation and fluorescence quenching in aqueous media 46,52-54.

Although PS-based FGS has attracted a lot of attention and has created several opportunities in the surgical oncological field, still the chances of misdetection of sub-centimeter tumors are frequent. This usually results in the emergence of lethal recurrent cancers which are more difficult to treat. A recent approach in developing more selective PS-based OI is based on the incorporation of tumor-specific bio-responsive elements with PSs known as aPSs or photodynamic molecular beacons. These photodynamic molecular beacons remain non-fluorescent and non-cytotoxic due to the linked quencher with the PS. Under tumor specific conditions the linker gets cleaved resulting in release of free active PS with restored fluorescence emission and ROS generation property. Therefore, upon irradiation, aPS-based theranostic agents enable better and controlled tumor-selective PS fluorescence at microscopic level imaging along with selective destruction of disseminated, microscopic tumor mass through PDT and/or other modalities like resection 22,27,34,55. Designing of photodynamic molecular beacons are based on three approaches: (1) aggregation-induced emission (AIE), (2) aggregation-induced quenching (AIQ) and (3) dual-labeled beacon 27,34. AIE based PDT beacons are weakly or negligibly fluorescent in dissociated free state but exhibit intense fluorescence and strong photosensitization in the aggregate state due to the phenomena of restriction of intramolecular motions (RIM) 56,57. While dual labeled PDT beacon is an advanced concept where the PS -quencher pair is conjugated with a tumor microenvironment bioresponsive linker molecule. Upon exposure to specific stimuli, disruptions of linker, lead to release of free PS regaining its fluorescence and ROS generation property 55,58.

The concept of PDT beacons dates back to 1982, when Moore et al., prepared carotenoporphyrin by joining a synthetic carotenoid to a tetraarylporphyrin through a flexible trimethylene linkage and showed that the carotenoid conjugation photo-protects the porphyrin and thus limit the nonspecific toxicity in biological systems 59. Following this, other carotenoid analog conjugated PSs like meso-tetraphenyl-substituted porphyrins, pheophorbide and Hp were investigated as selective fluorescence-based tumor imaging and PDT agents 60-62. However, this design failed to gain many applications due to the lack of selective separation and partial fluorescence quenching 63. In 2004, Chen et al., reported the first proof-of-concept of aPS beacon where pyropheophorbide was conjugated to ^1^O_2_ quencher carotenoid via a peptide linker, cleavable by a specific protease. Under *in vitro* condition this beacon also confirmed its selective activation upon targeting 64. Other groups have also reported other self-quenched PS beacons covalently attached to carbon nanotubes, commercially available black hole quenchers (BHQs) and other carotenoids 58.

As compared to the self-quenched PDT beacon, AIE PSs exhibit enhanced fluorescence intensity which allows improved fluorescence-based self-tracking PS distribution in cellular systems during PDT. Moreover, these AIE PSs have persistent ^1^O_2_ generation ability, which makes them potential candidates for efficacious fluorescence image-guided PDT. AIE PS theranostic molecular probes are designed with two main components: the AIE PS core, and the recognition element which is used to direct the *in vivo* PDT. Depending on the recognition groups the AIE PS molecular probes are classified as targetable and activatable. Designing of targetable AIE PS are based on the targetable ligand in tumor tissues where a choice of recognition element includes (i) hydrophilic targeting ligands such as biomarkers on the target cell surface; (ii) bioorthogonal labeling where the clickable groups are eventually interacting with the target cells via click reactions. Fluorescence and PDT activity of AIE PS is activated upon recognition interactions with the target ligand via the phenomena of RIM. While in the case of activatable AIE PS, the fluorescence and photosensitization property of PS is quenched by the tumor enzyme or stimuli-specific cleavable quenchers. Activatable elements provide improved selectivity of AIE PS molecular probes for imaging and therapy 57.

AIQ utilizes the concept of fluorescence quenching of PSs in aggregation state, thus keeps the fluorescence in the off state while circulation, and thereafter effectively regain their fluorescence upon disaggregation at the tumor site resulting into selective “off to on” fluorescence-based monitoring of tumors followed by PDT. The concept of AIQ based “off to on” fluorescence transduction has been demonstrated by Fu et al., where they reported a pH-responsive biodegradable and O_2_ self-supplying Mn^+2^ doped calcium phosphate mineralized glucose oxidase nanoparticles (NPs) loaded with catalase and sinoporphyrin sodium as PS. This study demonstrates a novel concept of starvation therapy enhanced PDT through the cascade reactions of glucose oxidase and catalase, whereby catalase catalyzes the decomposition of endogenous H_2_O_2_ to generate O_2_ which promotes glucose oxidase to consume more intratumoral glucose. Furthermore, O_2_ generation overcomes tumor hypoxia and improves PDT induced generation of ^1^O_2_. These NPs showed selective accumulation at tumor sites monitored by FLI as well as Mn^+2^ mediated MRI 65. In another study, Jiang et al., reported biocompatible polymeric NPs, pluronic F127 encapsulated hexyloxyethyl-devinyl pyropheophorbide-a (HPPH) and camptothecin (chemotherapeutic drug), whereby the NPs are selectively activated by ROS at the tumor site. Their “off-to-on” transition allowed tumor-selective FLI imaging along with synergetic chemo- and photodynamic treatment in *in vivo* xenograft tumor mice model 66.

Other approaches of designing activatable PDT beacons involve incorporation of multiple PSs to a polymeric carrier like chitosan, dextran, heparin, hyaluronic acid, glycol, polylysine, pullulan etc. Incorporation of PS in polymeric carriers results in efficient self-quenching of fluorescence quantum yield, thus nano-PS formulations remain photodynamically inactive during systemic circulation and activated only under tumor site specific molecular stimuli or environment conditions 58,67,68.

Over the decades, PS-based FLI-guided PDT and PDD had a long history of successful applications in clinical settings owing to high sensitivity, better spatial resolution, real-time and non-invasive mode of detection and visualization with already available instrumentations. However, due to off-target accumulation, activation, concentration and microenvironment-dependent fluorescence signal intensity of free PSs, limits their applications in real-time settings. These limitations of conventional PS have boosted the rapid advances in novel approaches and designing rationality for the development of next-generation PS-based FLI with the improved theranostic outcomes. A most feasible strategy that has been extensively explored includes the incorporation of PSs in nanostructures imparting the properties of targetablity, uniform tumor tissue distribution, higher stability and longer circulation time. Herein designing strategy based on assembly-driven molecular beacons and aggregations has been discussed as potential fluorescent imaging probes. The major importance of assembly-based PS design is that the photophysical properties of PS can be conveniently regulated by controlling their assembly arrangements. Furthermore, advances in imaging systems, and image analysis algorithms has also revolutionized the field of FLI-guided PDT. However, even after possessing many advantages most clinically and non-clinically used PSs are responsive to light of wavelength region 630 -700 nm with poor tissues penetration and their fluorescence properties vary considerably with a change in microenvironments, limiting the FLI resolution and sensitivity. Thus, the inherent limitations of visible region bioimaging have uplifted the research areas of new OI techniques in PDT such as PAI, SERS and UCLI, due to their unique strengths making them suitable for high-precision and resolution bioimaging.

#### Photoacoustic Imaging Combined Photodynamic Therapy

PAI (also referred as optoacoustic) is a rapidly emerging promising non-ionizing, non-invasive technique of biomedical imaging modality providing simultaneous *in vivo* structural, functional and molecular information at clinically relevant penetration depths. As compared to existing imaging techniques, PAI being a hybrid imaging method provides high spatial resolution and image contrast by combining the advantages of both OI (contrast quality, spectral sensitivity) and US imaging (in-depth tissue penetration, high resolution) 69,70. During PAI, absorption of light energy by endogenous chromophores (e.g., hemoglobin and melanin) present in tissues, results in their rapid thermoelastic expansion, generating wide-band photoacoustic (PA) transients or US waves. US transducers detect the generated US waves at the external surface of the tissue by converting the mechanical US waves to electrical signals which are further processed to form an image. Compared to conventional OI modality, PAI enables imaging at a greater penetration depth of tissues (5-6 cm), thus allows deep tissue imaging in clinics 70-72. PAI enables the imaging of dense and unorganized vasculature in malignant tumors due to the higher concentration of hemoglobin and melanin in tumor mass/vasculature compared with normal tissue. PAI have been reported to detect tumor vascular networks in the rat brain, the blood-oxygenation dynamics in the mouse brain, human arm, as well as breast cancer imaging based on endogenous chromophores 70. Additionally, utilization of PA exogenous contrast agents such as NIR-absorbing dyes (ICG, IRDye800CW and AlexaFluor 750), carbon nanotubes as well as gold NPs can significantly enhance the sensitivity and contrast of the PAI in deeply situated tumors 73,74. However, cytotoxicity issues and low blood circulation time of these exogenous contrast agents limit their clinical application 70,71. This necessitates the use of some biologically compatible agents with minimum side effects. In this regard Ho and colleagues evaluated five different PSs with low fluorescence quantum yields, zinc phthalocyanine (ZnPc), protoporphyrin IX (PpIX), 2,4-bis[4-(N, N-dibenzylamino)-2,6-dihydroxyphenyl] squaraine (Sq), chlorin e6 (Ce6) and MB as potential PA contrast agents in a phantom model. Among all the tested PSs, ZnPc showed the highest PA activity. Subsequent *in vivo* studies showed preferential accumulation of ZnPc in tumors which was evident from PAI of its localization and biodistribution thus enabling to achieve longitudinal monitoring of cancer. Thus, these PS-based PA contrast agents can offer great potential in PAI based cancer diagnosis combined with PDT. PSs with low fluorescence quantum yields usually possess high PA activity, due to the fact that an excited molecule can relax back to the ground state either through fluorescence emission or thermally through internal conversion 70. PS molecules can provide the advantage of combining FLI and PAI. Both imaging techniques provide a number of complementary advantages, FLI can gain sensitivity at single molecule level at superficial depth, while PAI accomplish significantly better deep tissue spatial resolution together with non-interference from photobleaching and autofluorescence issues 75. In another study, Abuteen et al., evaluated six free base tetrapyrrolic PSs of two different classes (quinoline-annulated porphyrins and bacteriochlorins) with NIR absorption, low fluorescence emission and ^1^O_2_ quantum yields for their PA contrast generating efficiency with respect to standard PA contrast agent ICG. The study demonstrated that these tetrapyrroles allow PAI of a sub-mm target up to 2 cm depth in tissue mimicking phantom, thus suggesting the use of these PSs as potential exogenous PAI contrast agents to be evaluated for *in vivo* studies 76. Similarly, Attia and colleagues investigated the biodistribution and fate of the phthalocyanines in the biological tissues by studying the PA activity of three water-soluble phthalocyanines: phthalocyanine tetrasulfonic acid (PcS4), Zn(II) phthalocyanine tetrasulfonic acid (ZnPcS4) and Al(III) phthalocyanine chloride tetrasulfonic acid (AlPcS4) in phantom and mice bearing oral squamous cell carcinoma xenograft. Among all the three phthalocyanines, PcS4 conferred the highest PA activity in both phantom and tumoral mice, showing the highest accumulation in the tumor at 1 h post-injection, suggesting PcS4 as a promising PA contrast agent and can be successfully exploited as a photodiagnostic agent 77.

Other than free base PS, many metallated and non-metallated PS-based nanoformulations have also been investigated as promising PAI agents. The optical absorption coefficient of a free base PS has shown to be not sufficient to obtain a significant SNR for *in vivo* PAI, because PA signal generation is mainly dependent on the optical absorption properties of the structure being imaged. Nanosystems with encapsulated PS molecules can provide enhanced PA contrast by quenching the fluorescence quantum yield of the closely spaced PS molecules 11. For example, MacDonald et al., showed that relative to free-base porphysomes, Mn-porphyrin-phospholipid provides enhanced PA signals with PAI in phantoms. This increase in PA signals is attributed due to a drastic decrease in PS excited-state lifetimes because of insertion of paramagnetic Mn^+2^ ions into porphyrins, thus directing the PS excited states into nonradiative decay paths and acts purely in the photothermal mode producing PA waves 78. Another porphyrin-lipid shell microbubbles (MBs), termed “porshe MBs”, were reported by Huynh and collaborators and were investigated for PA properties in tumor xenograft mice model. The dense packing of porphyrins within the shell of MBs displays very high optical absorption properties, generating strong PA signals, which was evident from the enhanced PA signals in tumor mass, post porshe MBs injections 79,80. A lipid-conjugated Zn-chlorin derivative was used to synthesize Zn-MeO-chlorin lipid nanovesicles which showed PA contrast enhancement with narrow NIR absorption band, which showed favorable spectral un-mixing within the biological light absorbing and scattering environment a hamster oral carcinoma model, thus proving its potential as contrast agents for PAI 81. Another study showed that due to endogenous light absorption by hemoglobin, only large blood vessels could be visualized in the tumor mice model upon PAI. However, enhancement of PA signal in the whole tumor mass occurred post injection of IR825@C18PMH-PEG-Ce6-Gd micelles, showing accumulation of these NPs in tumor tissue, thus allowing PAI-guided PDT 82. Similarly, PA image contrasting ability of the hypoxia-activated prodrug formulation AQ4N-hCe6-liposome was evaluated in tumor-bearing mice. PAI results indicated that mice those were intravenously injected with AQ4N-hCe6-liposome showed ~2.5 times increased PA signals in tumors than others without AQ4N-hCe6-liposome injection 83.

Furthermore, metallic plasmonic NPs exhibits several fold higher absorption coefficients compared to endogenous chromophores, making them favorable for PAI based monitoring for their tumoral uptake studies 11. As an example, Ce6 -loaded plasmonic vesicular assemblies of gold nanoparticles (AuNPs) reported by Lin et al., showed improved tumoral uptake of PS following heat‐induced release of the Ce6 cargo along with *in vivo* PAI combined PDT potential 84. Similarly, Ding et al., described the synthesis of nanocrystallite ZnPc nanodots (ZnPcNDs), by cryodesiccation-driven crystallization approach, which was further surface modified with Pluronic F127 and folic acid (FA), endowing them with good water solubility and stealth properties in blood, which allows them to avoid immunological detection and destruction thus prolonging their circulation time. These nanodots showed efficient cancer cell targeting, for simultaneous PAI and PDT 85. Also, ultra-small pyropheophorbide-a-PEG nanodots were reported for PAI/FLI-guided PDT with effective renal clearance properties without any long-term side effects 86. PS sinoporphyrin sodium loaded PEGylated graphene oxide reported by Yan et al., also exhibited great potential for enhanced FL/PA dual-modal imaging-guided synergistic PDT and photothermal therapy (PTT) in human adenocarcinoma mice model 87. Many other nanoformulations loaded with suitable PSs have been explored for PDT combined PAI theranostic agents 11.

Although PAI technology dates back to 1980s, however its being in recent years only that PAI have been explored extensively for its potential in optimizing PDT outcome by providing scalable and multi-contrast images with 3D real‐time feedback. PAI not only provides information about biodistribution, pharmacokinetics and uptake of PS in tumor mass for image‐guided PDT but have also been utilized for monitoring real‐time vascular destruction and changes in tissue O_2_ levels post‐PDT, thus allows optimization of PDT dose parameters. One of the important aspects is that even PSs with poor fluorescence quantum yield which are generally not suitable for FLI can be successfully utilized for PAI. As discussed, designing of nano assemblies of PSs leads to novel approaches of highly spatiotemporal and non-invasive combination of FLI and PAI. However, various instrumental and biological conditions limit PAI's performance in the clinical set-up which includes (1) limited aperture and finite size and shape of US transducer results into “limited-view” condition, (2) narrow detection bandwidth of transducer compared to a wide frequency range of PA signals generated from the sample surface causes bandwidth mismatching, (3) a part of optical and acoustic attenuation effects in tissues also results in a poor spatial resolution which further decreases with depth and (4) tissue heterogeneity cause a complex speed-of-sound distribution that complicates image reconstruction 88. Thus, utilizing PS nanoconstructs for PAI holds great promise into clinical translations provided that the limitations of instruments and image reconstruction algorithms need to be addressed.

#### Upconversion Luminescence Imaging Combined Photodynamic Therapy

**Figure 6:**
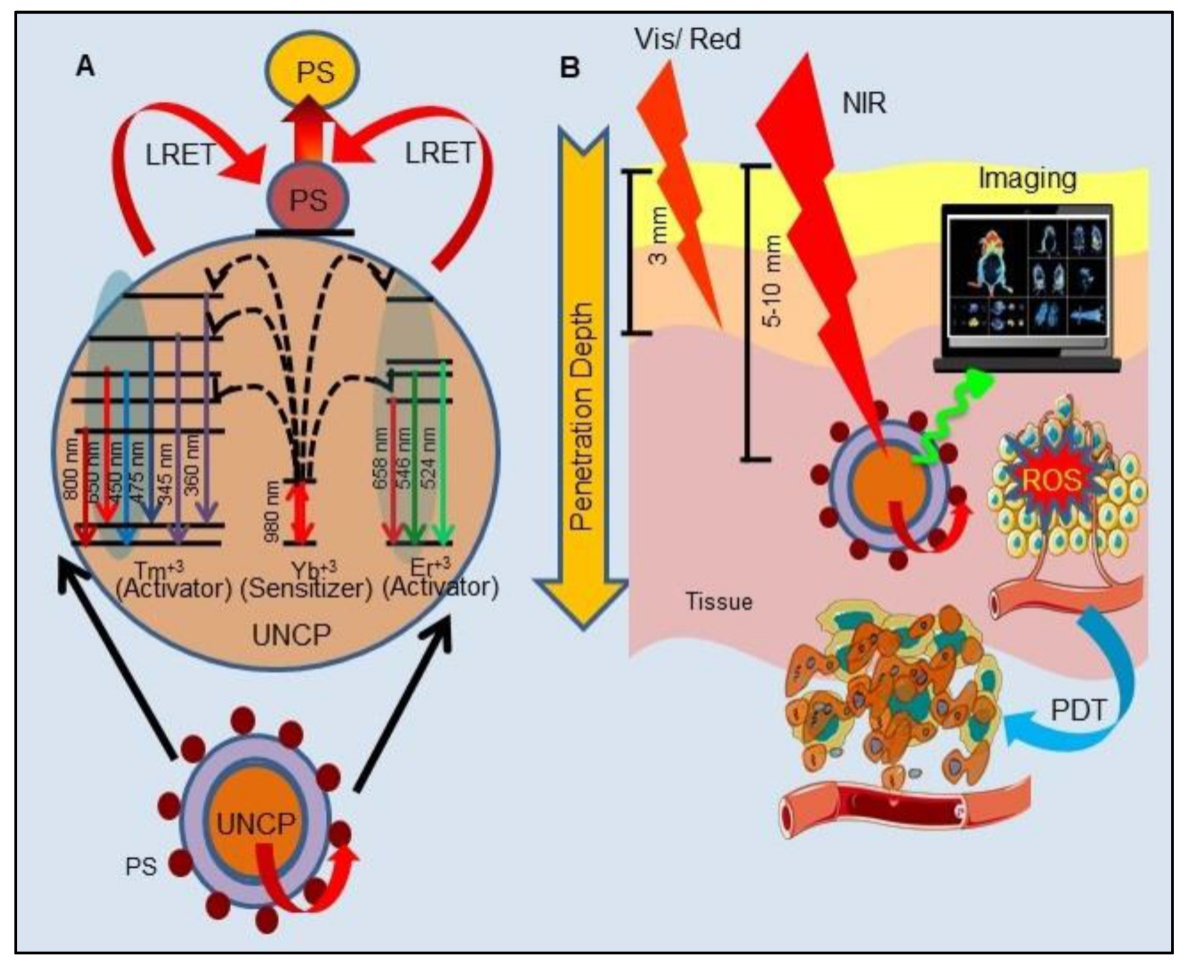
** Schematic illustration of principle of upconverting nanoparticles (UCNPs) mediated Upconversion Luminescence (UCL) imaging and Photodynamic Therapy (PDT): A.** Upconversion process in the UCNPs under Near Infrared Radiation (NIR) excitation, and the Luminescence resonance energy transfer (LRET) between UCNP and photosensitizer (PS). **B.** Deeper penetration of NIR compared to visible light excites UCNPs and converts NIR to visible wavelength emission for activation of the PS producing Reactive Oxygen Species (ROS) to induce PDT mediated tumor damage with simultaneous imaging with UCL.

In recent years, UCL has gained notable attention in the field of cancer therapy and imaging over conventional luminescence techniques (organic dyes and quantum dots). In principle, UCL is an optical process and make use of lanthanide ions (Yb^3+^, Er^3+^, and Tm^3+^) based upconversion nanoparticles (UCNPs) which are excited by low-energy radiation (NIR light) to generate higher energy emission (visible or UV light) caused by an anti-stokes mechanism (Figure 6A). In comparison to conventional light responsive fluorescent imaging agents, UCNPs possess many unique properties of high conversion efficiency and light penetration in biological tissues such as photostability, long lifetime, non-photobleaching, narrow emission peaks, large stokes shifts and low toxicity. Moreover, the use of low energy radiation reduces light induced unwanted cellular damage and offers the advantages of very low auto-fluorescence and high detection sensitivity. All these advantages widened the application of UCNPs from therapy to *in vivo* imaging 89. UCNPs have already been utilized to overcome the limitation of visible light excitation of traditional PSs, which suffers from low penetration depth in tissue to achieve NIR-triggered PDT. Majorly, lanthanide-doped UCNPs have shown to accomplish NIR-triggered PDT. As illustrated in Figure 6A, upon excitation by NIR, UCNPs efficiently convert the deeply penetrating NIR into visible wavelength light which eventually excites the UCNP conjugated or encapsulated PSs, bestowing the essential advantage of PDT of deep-seated tumors. Furthermore, excitation of UCNPs with NIR light results in release of photons of both higher wavelength (red) and shorter wavelength (green), which makes them a potential candidate for simultaneous PDT and *in vivo* OI or more specifically UCLI-guided PDT (Figure 6B) 89-92. For example, Park et al., reported the synthesis of Ce6 conjugated NaYF4:Yb,Er/NaGdF4 core-shell, which showed accumulation in tumor tissues by the enhanced permeability and retention (EPR) effect in the tumoral mice model. Upon excitation with a 980 nm laser, UCNPs showed both red and green emission, used to visualize tumors with UCLI and also demonstrated efficacious PDT outcomes 93. Similarly, excitation of MC540-AuNR@UCNP@NBUCL with 808 nm laser allowed high-resolution UCLI of tumors by upconverted green and red lights at 520 nm and 654 nm along with AuNR@UCNP energy transfer mediated PDT by Merocyanine 540 94.

Another bioresponsive FeOOH modified and toluidine blue (TB)-loaded NaLuF4:Yb,Er,Tm@ NaLuF4 (RE-FeOOH-TB) nanoformulation was reported for UCLI-guided PDT for *in vivo* tumor model. In response to intratumoral acidity and reducibility, FeOOH reacts and quenches GSH, subsequently releasing Fe^2+^ and catalyzing H_2_O_2_ to produce O_2_, which improves intratumoral dissolved O_2_ for PDT. Upon NIR excitation, UCNPs emits 800 nm mediated UCLI and 650 nm for excitation of TB for PDT 95. Similarly, many other PS conjugated UCNPs, such as hyaluronate fullerene conjugated 3-aminophenylboronic acid functionalized UCNPs, ZnPc conjugated spindle UCNP@SiO_2_@AuNPs, trismethylpyridylporphyrin-fullerene (PC70) decorated FA/PEG-coated NaGdF4:Yb,Tm@NaGdF4, ZnPc conjugated NaYF4: Er@NaXF4, AlPcS4-conjugated PEG-coated Fe_3_O_4_@NaYF4:Yb/Er have been shown to exhibit promising PDT efficacy combined with UCLI 96-100. Recently Feng et al., introduced proof of concept to realize PDT “off” and “on” switching, to overcome the damage to normal cells and the photosensitivity to the skin during imaging/diagnosis by making use of bioorthogonal chemistry. They utilized UCNPs anchored with one handle of click reaction tetrazine (Tz) and targeting entity to form UCNPs-Tz/FAPEG. UCL images of tumor region were recorded after intravenous (i.v.) injection of the UCNPs-Tz/FAPEG upon exposure to 980 nm laser light which showed their selective accumulation in tumor tissue due to FA targeting and EPR effect. Upon selective accumulation, *in situ* injections of the other handle of the bioorthogonal reaction, Rose Bengal (RB) conjugated norbornylene (NB), allowed click reaction between Tz and NB to efficiently link the PS to the UCNPs. This further enabled highly effective PDT in tumor-bearing mice via effective energy transfer between UCNP and the RB 101.

Owing to several advantages of UCNPs, UCLI is being considered as an upgraded alternative to traditional OI-guided tumor therapy more importantly for their ability in long-term repetitive FLI and effective penetration depth in deep-seated tumor by NIR excitation. Furthermore, UCNP acts as suitable tumor-selective drug delivery carriers for hydrophobic PSs along with activating PSs in deep-seated tumors for effective PDT. Further, as wavelengths of > 800 nm are used for UCNP-PS excitation, this is advantageous over the use of free PS molecules. Far-red wavelength of 800 nm cannot be used for excitation of free PS, as the photons of wavelengths of ≥ 800 nm do not provide enough energy to excite O_2_ to ^1^O_2_ state. Another interesting feature is the utilization of the same lanthanide doped UCNPs for multimodal imaging capabilities for example NaGdF_4_ for MRI and UCLI; NaLuF4 for CT and UCLI and radionuclide UCNPs for UCLI combined PET/SPECT. Moreover, PA contrast agents absorbing in NIR region causes an increase in the PA signal thus UCNPs can also be effectively exploited for dual PAI and UCLI. Although NIR have deep penetration in tissues, however considering the fact that the emitted UCL must be detected outside the imaging object, UCLI penetration depth was reported to be only ~3.2 cm upon 980 nm excitation 102. Besides this, certain other caveats include (1) NIR excitations (~ 980 nm) generates non-negligible heating of the exposed tissues and also cause light attenuation due to overlap with water absorption; and (2) narrow excitation bands and low absorption cross-sections of UCNPs together with surface quenching effects reduce UCL brightness 103. Further, the practical applications and designing of UCNPs for UCL guided PDT impose several challenges majorly in their designs such as their composition, size, surface functionalization, physiological stability, PS loading, upconversion efficiency and absorption spectra overlap of UCL-PS pair 91. Thus, multidisciplinary efforts need to be addressed for establishing this synergistic combination of UCNP and PSs to augment the scope of improved PDT and imaging in near future.

#### Surface-Enhanced Raman Scattering Imaging Combined Photodynamic Therapy

**Figure 7:**
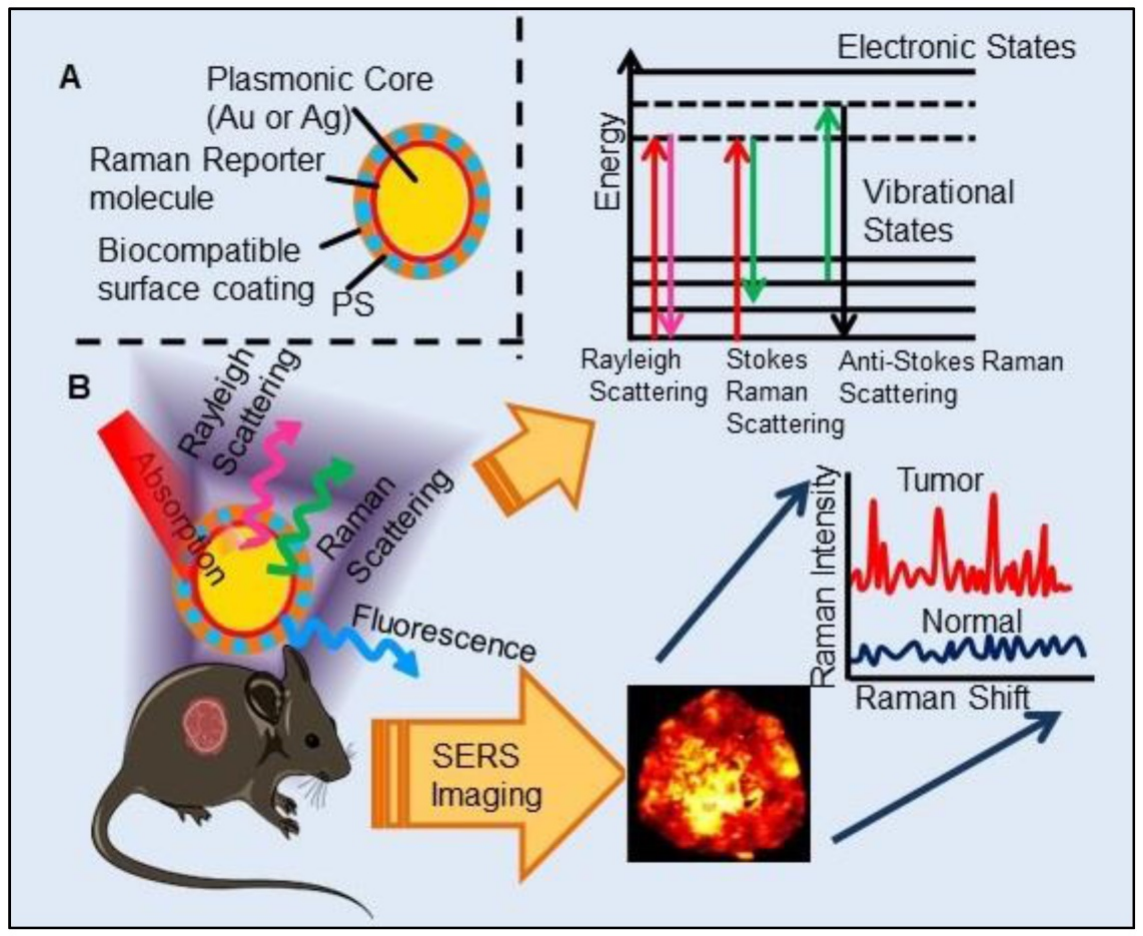
** Schematic design and illustration of Surface-Enhanced Raman Scattering (SERS) probes for *in vivo* imaging: A.** Structure of SERS probe consisting of a metal nanoparticle as plasmonic core, adsorbed Raman reporter molecule on the metal surface, a biocompatible surface coating layer loaded with photosensitizer (PS), **B.** Depiction of energy transitions of photons during different types of light scattering upon absorption of light by plasmonic nanoparticle. Representation of SERS image and SERS spectra of tumor.

In past few years, SERS imaging with biocompatible SERS probes have emerged as a novel *in vivo* cancer imaging technique. SERS is a phenomenon in which the Raman intensity of a molecule is enhanced enormously (up to 10^14^-fold) when placed near a noble metal surface such as silver or gold NPs. SERS imaging holds great potential over other imaging methods in the field of medical imaging as the SERS nanoprobes have enhanced photostability, high sensitivity, higher signal specificity and multiplexing capabilities 104. As illustrated in Figure 7, the principle of SERS imaging is based on electronic transitions of both Rayleigh and Raman scattered photons by SERS probe, further having frequency and wavelength different from the incident photons. SERS imaging has also proved to be efficient in the detection of circulating tumor cells and multiplex tumor-associated cell surface antigens and can be used to visualize tumor margins allowing guided tumor resection 105. Interaction of photoactive molecules, porphyrins with metallic nanostructures imparts several important properties which include charge transfer, plasmon-enhanced electrical conduction and electrocatalytic activity. The heterocyclic pyrrole structure of porphyrins contributes to its strong Raman scattering properties, and which can be conveniently enhanced by both electromagnetic and charge transfer to exhibit SERS spectra. Further the absorption spectra of porphyrins have significant overlap with the plasmon band of AgNPs and forms charge-transfer complex with AgNPs, thus making porphyrins potential SERS imaging agents combined with PDT. As an example, the complexation of tetra(4-aminophenyl) porphyrin with AgNPs shown to undergo charge-transfer complexation in the ground state, which was confirmed by red-shifted ground-state absorption and SERS property 106. Interestingly, Zhang et al., reported the interaction of RB with silver island films (SiFs) caused a three-fold increase in ^1^O_2_ generation due to the enhanced triplet excited state yield of the PS, a phenomenon known as Metal-Enhanced ^1^O_2_ generation resulting from the interactions between plasmons and photoactive molecules 107. Similarly, many other PSs such as 5-ALA, PpIX, conjugated on AuNP surface significantly enhanced the PDT efficacy in cancer cells which is attributed to the enhancement in ROS generation by PS due to highly localized plasmonic field of the AuNPs 108,109. These findings formed the basis for the development of novel theranostic systems for SERS imaging-guided real-time monitoring of PDT. In most of the reported studies, AuNPs have been widely used as SERS imaging probes and photothermal agents due to their enhanced SERS effect and high photothermal conversion efficiency 105,110-113. While very few studies have involved SERS-PS probes for PDT combined SERS imaging. One such example involves FA receptor mediated SERS imaging, based on the utilization of nanodrug PpIX-GNR-MBA-FA. Where, PS PpIX function in SERS imaging, mecaptobenzoic acid (MBA) as Raman reporter molecule and Gold nanorods (GNR) mediates photothermal conversion and enhanced PDT efficiency 114. Other studies include multilayer-coated AuNPs where PSs (PpIX, MB) were loaded in silica and polymer layers, they showed potential for simultaneous SERS-based tumor detection and PDT in the tumoral model 115,116. Zheng et al., demonstrated a novel strategy to construct SERS probes by utilizing a non-fluorescent Mn-porphyrin-phospholipid conjugate (MnPL) to serve as both the Raman dye and a stabilizing biocompatible surface coating agent on AuNPs 117. Further, they coated AuNPs with other non-fluorescent Pd-porphyrinoids forming PdPL-NPs where coupling with plasmonic NPs imparted both SERS mediated reporting and monitoring capability. Interaction between plasmonic metallic NPs with fluorescently inactive PSs allow for the decoupling of the therapeutic and imaging mechanisms so that both phenomena can be optimized independently. This phenomenon allows for *in vivo* tracking of non-fluorescent PSs which is otherwise not possible with conventional FLI along with overcoming its limitation of background autofluorescence. Most importantly, this design allowed the excitation of PdPL-NPs with the same laser wavelength (638 nm) to stimulate them both as SERS reporters and photosensitizing agents. This provides the advantage of real-time SERS-based dosimetry for monitoring PS concentration at the tumor site and PDT dose delivery. Moreover, nano-enabled SERS reporting PSs uses entirely different physical mechanisms of absorption by PSs and scattering by AuNPs, resulting in the mutually exclusive output of enhanced PDT and SERS signals 118.

Although the results obtained with PS based SERS reporter agents are fascinating, still this research is at the basic stage and more in-depth understanding of the underlying mechanism(s) governing the change in SERS intensity in relation to the PDT dose and other more efficient designs needs to be explored. In general, the designing of SERS nanoparticles is based on certain logical approaches: (1) the selected metallic material should have high SERS enhancement, which restricts the utilization of only three best-known SERS materials Au, Ag and Cu, (2) encapsulation of SERS nanoparticles to protect and isolate the Raman active material from surrounding *in vivo* conditions which is most important to preserve the unique identifying Raman fingerprint and retain its detection ability 119. Despite the advantages of SERS imaging, other than rationale designing of SERS, many additional technical obstacles need to be addressed before its clinical deployment. Effort needs to be taken for developing more advanced Raman detectors with a wide field of view, rapid image acquisition speeds and deep tissue imaging ability as the presently available Raman scanners lack all of these. Further, development of more robust imaging algorithms are required to detect very fine spectral differences between cancerous and non-cancerous cells, which usually goes undetectable in direct analysis of the Raman spectra.

### Magnetic Resonance Imaging combined Photodynamic Therapy

Since its first application, MRI has become one of the powerful, non-invasive technologies for soft tissue imaging in clinics, among all the available oncological diagnostic methods. It enables 3D anatomical imaging combined with high spatio-temporal resolution thus provides the ability to track physiological and molecular events. The contrast generated due to magnetic resonance (MR) signal occurs due to differences in relaxation times of water protons (T1 and T2) and/or the proton density of water molecules in different soft tissues. These differences in proton behaviors allow tissue discrimination 120. Although in most of the cases, the contrast generated due to indigenous tissues protons between healthy and diseased tissues are too weak to be imaged which necessitates the use of external contrast agents. These contrast agents generally enhance the relaxation behaviors of interacting water protons in its vicinity, thus improving the overall MRI image contrast. MRI contrast agents are either paramagnetic ion complexes or superparamagnetic magnetite particles (iron oxide nanoparticles, SPIONs), which are being used for cancer detection and diagnosis. Paramagnetic complexes containing manganese (Mn^2+)^ or gadolinium (Gd^3+^) tends to reduce both T1 and T2 relaxation times, while SPION contrast agents shorten the T2 and T2* relaxation times of the residing tissues. Based on the differences in generated MRI weighted images, paramagnetic and SPION contrast agents are known as positive and negative contrast agents, respectively 121. Gd(III)-based chelation complexes such as GdDOTA (DOTA = 1,4,7,10-tetraazacyclododecane-1,4,7,10-tetraacetate), GdDTPA (DTPA = diethylenetriamine pentaacetate) are routinely used in clinics 121. MRI has already shown its promising applications in the detection of solid tumors for image-guided local destruction of cancerous tissues by various therapies like radiotherapy, radiofrequency (RF) ablation, thermoablation, cryoablation, and laser ablation. Similarly, contrast-enhanced MRI-guided PDT, has been explored successfully for precise detection of interstitial lesions and guide local light irradiation to maximize the therapeutic efficacy 122*.* Further, studies have shown that PDT-induced changes alter proton behaviors in the tumor tissue and such changes can be enhanced using MRI contrast agents. Thus, MRI-guided PDT allows the determination of treatment parameters like PS concentration, light dose in relation to the final treatment outcome 32.

Administration of Gd-based MRI contrast agents post-PS injection and PDT have already been shown to provide various sensitive information for the assessment of treatment outcome along with direct visualization of vascular damage for *in vivo* MRI-guided PDT in humans 21,32. Although administration of contrast agent and PS as separate entities imparts the limitation of different pharmacodynamics and pharmacokinetics of two agents, resulting in differences in their biodistribution-based imaging. Incorporating a MRI contrast agent and a PS in a single theranostic agent has shown to overcome this limitation where the contrast agent and PS having the same biodistribution patterns further optimize the PDT treatment. Moreover, such a strategy of combining bimodal agents within a single molecular entity provides with the further advantages of increased hydrophilicity of PS in biological fluids and improved proton relaxivity for MRI efficacy due to the increased molecular weight 120. Moreover, increasing interest in designing an efficient PS based contrast agents lies in the fact that till date no commercially available contrast agents are efficient enough for effective detection of malignant neoplastic tissues. Designing of potential contrast agents are based on various crucial factors like kinetic and thermodynamic stability, binding efficiency of water molecules with the complex, its exchange rate with the bulk water system, strength of the magnetic field, effect of temperature, and concentration 37. Different approaches for developing MRI-PDT theranostic agents involve PS-MRI contrast agent as conjugated complexes, paramagnetic metallo-PSs and nanoformulations of contrast agent and PS. Although most of these PS based contrast agents have not been explored for their PDT ability, still these bi/multifunctional complexes have the potential for combined MRI and PDT applications.

#### Paramagnetic Metallated Photosensitizers

Paramagnetic metallo-PSs are the most extensively explored approaches for designing contrast agents. Incorporation of paramagnetic metals within tetrapyrrole rings of porphyrins along with appropriate side groups have been shown to enhance the relaxivity of surrounding water molecules thus generating MR contrast. Further, these complexes have a lesser possibility for release of metal ions from the porphyrin structure during systemic circulation and tissue accumulations due to their kinetic inertness, which lowers the risk of systemic toxicity induced by Gd and Mn ions 37. The potency of metallated tetrapyrrolic-based macrocycles were explored as MRI contrast agents for the first time in 1984. First PS based contrast agents were based on Cu(II), Fe(III), and Mn(III) inserted meso-tetrasulphonated phenyl porphyrin (TPPS4). Among these metallo-TPPS4 complexes, Mn(III)-TSPP4 was shown to be the best MRI contrast agent, followed by Fe(III)-TSPP4, while Cu(II) complexed TSPP4 showed the least MRI contrast 123. In 1984, Furmanski et al. demonstrated the paramagnetic metalloporphyrins as tumor-selective MRI contrast agents for *in vivo* imaging of human tumor xenografts in nude mice 124. Among all other metallated-PS complexes, Mn-meso-sulfonatophenyl porphyrins have drawn attention as potential MRI contrast agents with a variety of different formulations, designs or functional groups and investigated in various *in vivo* animal cancer models 125-127. Another study reported that a water-soluble C60-Mn porphyrin exhibited improved water protons relaxation in comparison to free Mn-porphyrins due to the introduction of fullerene derivatives to Mn(III) porphyrins 128. Furthermore, Mn-porphyrin complex has also been studied as partial oxygen pressure (pO_2_)-responsive MRI contrast agents based on the redox switching of Mn(II/III)-porphyrin complexes 129.

Besides transition metals, porphyrin cavity can also accommodate lanthanide ions, which are of potential interest in a range of diagnostic and therapeutic medical applications. Due to the large size of Gd^+3^ ions, it cannot get accommodated in the macrocyclic center of common porphyrins, hence a new class of expanded porphyrins known as texaphyrins were used to synthesize Gd(III)- PS complex 130. Sessler et al., synthesized and investigated a water-soluble, stable and non-toxic Gd(III)-texaphyrin/ Motexafin gadolinium (MGd) as a promising *in vivo* MRI contrast agent capable of targeting the liver and useful for detecting cancers, with 3-4 times greater relaxivity than the conventional MRI contrast agents can generate 131,132. Although, Gd(III)-texaphyrin intensively studied for cancer PDT or radio-therapy from preclinical to clinical trials, however, it raised concerns due to disappointing results and controversies about the reproducibility of studies 130. Similarly, two porphyrazine macrocycles complexes chelated with central Gd^+3^ cation, were reported as multifunctional agents for MRI and FLI-guided PDT 133.

#### Photosensitizer-MRI Contrast Agent Conjugates

Another approach of developing tumor-avid MR contrast agent involves conjugation of already available MRI contrast agents with the tetrapyrrole structure of PS as functional side groups. Such PS-MRI contrast agent conjugates provide several advantages such as (1) increased relaxivity of contrast agent due to the increased size of the conjugates, promotes better proton exchange property along with reducing the tumbling rate of the molecule; (2) hydrophilic contrast agent confers amphipathicity to the conjugates, helps in increasing the water solubility of hydrophobic PS and promotes accumulation in the tumor site 134. One of the first such examples is bis-Gd-DTPA-mesoporphyrin (Gd-MP or Gadophrin-2), where two Gd-DTPA moieties have been covalently linked to mesoporphyrin as side chains 135. Later, Gadophrin-2 was modified into Gadophrin-3 by inserting a copper at the porphyrin core of Gadophrin-2 which improved the stability and safety of the complex 130,136. Upon MR imaging, both the complexes showed greater accumulation in necrotic tumor regions in comparison to viable tumor tissue in xenograft tumor mouse models 135,137. Moreover, Gadophrin-2 was studied as a bifunctional contrast agent for *in vitro* cell labeling and *in vivo* tracking of transplanted stem cells using fluorescence microscopy, OI and MRI 138. In some porphyrin-paramagnetic ion/complexes, chelation has been carried out at both core and side groups to improve proton relaxation efficacy, for instance Mn-methylpyrroporphyrin-gadopentetate di-meglumine (Mn-MPP-Gd-DTPA) 130,139. The core structure of 5,10,15,20-tetraphenylporphyrin (TPP) was linked to one and four GdDTPA complexes through amide bond formation as side chains, which showed better MR relaxivity, compared to that of the widely used Gd-DTPA 140. In an effort to combine two or more different diagnostic imaging techniques, Gros et al., synthesized a series of water-soluble bi/multimodal heterobimetallic contrast agents, where MRI agents like gadolinium polyaminopolyacetates (Gd(DO3A-AM) and [Gd(DOTA)(H2O)] are linked as side chains on a Cu-porphyrin. These heterobimetallic Gd(III)/ Cu(II) complexes imparts MRI contrast by Gd(III) agents, while, PET, OI and FLI due to ^64^Cu-porphyrin 141,142. In another study, Zn(II) and Cu(II) porphyrazine (Pz) have been linked to GdDO3A-amide analogs, where Cu-Pz-Gd(III) complex exhibited the highest relaxivity, better cellular internalization and MR contrast enhancement, representing necrosis both *in vitro* and *in vivo* 143. Designing of PS-MRI contrast agent conjugates is mainly focused on the use of Gd(III) ions due to the superior paramagnetic character of Gd^3+^ ions. Thus, researchers prefer to obtain Gd-PS based MRI agents by covalently linking Gd-MRI chelates at the periphery of tetrapyrrolic macrocyclic core 36.

Several PS-contrast agent conjugates have been explored as multimodal theranostic agents for combined diagnostic imaging (MRI and FLI) and PDT applications 36. Although utilization of tetrapyrroles as tumor avid MRI contrast agent has been started early, however, applications of these complexes as bi/multimodal theranostic agents were investigated lately. In 1993, Hindré et al., studied a series of Gd(III)DTPA conjugated TPP, which showed promising results for both MRI and PDT in nude mice 144. This was reported to be the first prototype used for combined MRI-PDT theranostic applications. Later in 2000, Pandey et al., extensively investigated a new family of bifunctional theranostic agents combining two modalities into a single cost-effective “see and treat” approach, i.e., a single agent that can be used for contrast agent-enhanced MR and FLI followed by targeted PDT 145,146. Investigated agents were based on a chlorophyll-a derivative HPPH, conjugated with one, two, three, or six GdDTPA. HPPH-3GdDTPA was reported to be the best candidate with respect to its imaging and PDT efficacy 32. Similarly, phthalocyanine and porphyrazine - Gd(III) contrast agent conjugates also showed promising results for cancer imaging with both MRI and NIR fluorescence, along with effective phototoxicity 147,148. In an effort to integrate chemotherapeutics and PDT along with MRI, Wu et al., synthesized tetraplatinated porphyrin Gd(III) complexes (Gd/Pt-Porphyrin) which was simultaneously conjugated with cisplatin at the side groups and Gd(III) to the central cavity of tetra-(4-pyridyl) porphyrin. Herein, the platinum component in Gd/Pt-Porphyrin induced chemotherapy while the Gd(III)-porphyrin complex played dual roles as MR imaging and PDT agent 149. Ventura et al., synthesized conjugates with four [GdDTTA] linked to a meso-TPP core, as well as its yttrium(III) analog, as potential theranostic agents for MRI and one photon PDT. Further, Ventura et al., reported a series of conjugates with diketopyrrolopyrrole-Zn(II) porphyrinic core linked to a varied number of GdDOTA moieties; DPP-ZnP-GdDOTA, DPP-ZnP-(GdDOTA)2 and GdDOTA-ZnP-ZnP-GdDOTA. These conjugates exhibited good ^1^O_2_ generation ability with high NIR two‐photon PDT in cancer cells with remarkable fluorescence and MR imaging property. Two-photon PDT involves excitation of PS at the NIR region, which allows deep tissue treatment with minimal photodamage to surrounding healthy tissues. Further, two-photon irradiation can achieve high spatial treatment precision due to its application in a very restricted area 134,150,151.

#### Photosensitizer-conjugated MRI Nanoparticles

Recently to overcome the limitations of solubility and stability of PSs in blood circulation, photosensitizing NPs has emerged as a better therapeutic strategy to improve PS tumor targeting. Moreover, encapsulation of PSs in NPs allows for multi-modal/functional PDT-based synergistic therapeutic and diagnostic applications with improved theranostics 152. PS-NPs have also been applied for MRI applications. In a simpler approach, a PS-MRI contrast agent Gd(III)-meso-tetrakis(4-pyridyl) porphyrin has been conjugated with chitosan NPs by passive adsorption, and investigated for its MRI contrast agent property 153. While more complex nanoformulation designing involved multifunctional NPs of DOX@PLA@Au-PEG-MnP, which was fabricated of gold nanoshell surrounding doxorubicin (DOX) entrapped poly (lactic acid) core. Mn-porphyrin derivatives were coated on the surface of the gold shell through PEG linkage. Herein the gold nanoshell acted as NIR photon absorber, DOX as chemotoxic drug and Mn-porphyrin for PDT and MRI. These NPs exhibited synergistic chemo and photo-therapeutic effect and facilitated the imaging of locations and detailed structure of the tumor in nude mice through MRI 39,154.

Another strategy for designing nano PS-MRI contrast agents involve paramagnetic-porphyrins coated on magnetic particles core. Huang et al. strategically designed multifunctional PS based theranostic NPs where Ce6 was covalently linked on the surface of magnetic NPs (MNPs). Further during the investigation, Ce6-MNPs were observed to preferentially target tumors by application of external magnetic fields in gastric cancer mice model. Thus, this study demonstrated the successful design of targeted multifunctional magnetically guided PS delivery formulation as potential candidates with *in vivo* dual-mode NIR/FLI and MRI-guided PDT 155. Similarly, HSA coated MNPs loaded with bacteriochlorin, porphyrin linked silica-coated MNPs, TPPS4 coated water-soluble Fe_3_O_4_ MNPs, self-assembled polymeric nanocapsules with dendrimer porphyrin incorporated in SPIONS, dextran-coated MNPs bearing porphyrin, ZnPC encapsulated silica-coated MNPs based porous microspheres and many other such nano-formulations have been evaluated for MRI-guided PDT 36,39,156,157. MacDonald et al., reported the development of an organic, photonic NP, Mn(II)-porphysome (porphyrin-lipid) as potent MRI-photothermal NP, also capable of PTT and PA tomography 78.

Contrary to inorganic NPs, theranostic protein NPs have also been explored for cancer imaging and therapy, but with a very limited successful outcome. Few protein-based NPs utilizing viral capsid protein structure and other human proteins have been explored with PS for MRI and PDT applications. Paramagnetic Mn(III) Pp IX complexed with bacteriophage P22 capsid template and cowpea chlorotic mottle virus (CCMV) protein cage NP incorporated with Gd(III)-DOTA ligands and ZnPc have been explored as MRI contrast agents along with their potent PDT efficacy 158,159. While in another work, Zhou et al., investigated size-tunable Gd_2_O_3_@albumin conjugating Ce6 (GA-NPs) using hollow albumin as the nanoreactor for MRI-guided photo-induced therapy 160.

Overall, MRI is considered to be one of the significant molecular imaging modalities owing to its excellent temporal and spatial resolution of micrometers with non-invasive methodology, unlimited tissue penetration, non-exposure to ionizing radiation, high soft tissue contrasts together with 3D, real-time imaging. However, MRI suffers from poor sensitivity, high cost, slow image acquisition, long post-processing times and administration of higher concentration of contrast agents 161. Moreover, various factors need to be considered for application of metalloporphyrin-based conjugates and NP materials as a potential contrast agent for MRI. Rationale designing of PS-MRI contrast agents with enhanced relaxivities, increased contrast effect and brightness, stability against demetallation under physiological conditions and target specific biodistribution is highly desirable research area.

### Positron Emission Tomography/ Single Photon Emission Computed Tomography Imaging combined Photodynamic Therapy

PET/SPECT, radiotracer-based noninvasive imaging techniques have gained extensive interest in the field of cancer diagnostics. PET imaging which is based on the radioactive decay of positron-emitting isotopes, provides functional, metabolic and molecular assessment of normal and diseased tissues 146. PET radiotracers emit a positron (a positively charged electron) which in turn interacts with a surrounding free electron causing an annihilation reaction. This annihilation reaction results in the simultaneous production of two 511-KeV gamma rays (photons) at ~180° to each other which are further detected by the PET camera to generate an image. PET with 2-deoxy-2-[fluorine-18]-fluoro-D-glucose (^18^F-FDG), an analog of glucose, is the only PET probe approved by the Food and Drug Administration (FDA) for diagnosing, staging, restaging and detecting recurrence and treatment outcomes of various cancers to guide patient care in clinics. ^18^F-FDG, provides valuable functional information based on the increased glucose uptake and glycolysis of cancer cells and depicts metabolic abnormalities 162. ^18^F-FDG-PET allows planning, therapeutic response monitoring to PDT along with real-time monitoring of PDT mediated systemic transient metabolic changes in cells (apoptosis, necrosis, proliferation, metabolism) and vascular damage 163,164. Although ^18^F-FDG is an effective tumor-localizing tracer, it is not tumor-specific. Moreover, the short half-life of ^18^F-isotope (110 min) limits its use in PDT studies, due to considerably longer time of PSs to both accumulate in tumors as well as eliminate from the non-targeted organs 146. Thus, this necessitates the use of radiolabeled PS to evaluate the PS biodistribution itself. Similar to the simple chemistry of formation of metallo-PS complexes, PS can be radiolabeled via simple complexation chemistry, due to their metal chelation property 165. Radiolabeling of tetrapyrroles with Cu-64 for the localization of brain tumors and to develop coincident scintillation counters was first reported in 1951. Still, thereafter due to the technical limitations of earlier PET scanners, efforts to investigate the utility of ^64^Cu-labeled PSs as PET probe were very disappointing. It was only in the 1980s, when Wilson et al., attempted to detect brain tumors using ^64^Cu-Hp for *in vivo* measurements of its tumor uptake through PET imaging. However, the results of this study failed to gain interest, due to the poor tumor localization ability of ^64^Cu-Hp and the poor spatial resolution of PET scanners at that time. Nevertheless, this study provided a valuable result that ^64^Cu-labeling did not alter the main characteristics (biodistribution, pharmacokinetics, clearance) of the host PS molecules 166-168.

Among all the available PET radioisotopes, ^64^Cu labeled tetrapyrrole PSs especially porphyrins have been extensively studied as promising PET probes, because of the ability of tetrapyrroles as ^64^Cu chelator. Furthermore, photophysical properties and tumor-specific uptake of the macrocyclic PSs, long half-life of the Cu-64 isotope together with their biological compatibility, thermodynamic and kinetic stability made ^64^Cu- PS complexes an attractive option not only for PET as well as for therapeutic applications 169.

A ^64^Cu labeled metal-free sulfonated metallophthalocyanines ^64^Cu-CuPcS was evaluated for biodistribution studies in tumor-bearing rats using small animal-PET. However, the results showed most of the ^64^Cu activity within the kidneys and liver, with low uptake in tumor 170. Similar effects were found in studies with other Cu-64 labeled PSs such as ^64^Cu-5,10,15,20-tetrakis(penta fluoro phenyl) porphyrin, ^64^Cu-labeled tri and tetra sulphonated phthalocyanines 171,172. Rong et al., developed HPPH loaded PEG-functionalized graphene oxide (GO), forming GO-PEG-HPPH complex. Following radiolabeling of HPPH with ^64^Cu, *in vivo* biodistribution and delivery were tracked by PET, revealing increased tumor uptake of the complex resulting in improved photodynamic cancer cell killing efficacy 173. Shi et al., demonstrated a ^64^Cu labeled pyropheophorbide-α-peptide (GDEVDGSGK)-folate (^64^Cu-PPF) as a targeted PET imaging probe for folate receptor (FR)-positive tumors along with fluorescent/PDT property. Selective uptake of ^64^Cu-PPF in FR positive tumors was evident from high tumor-to-muscle ratio of 8.9, observed 24 h post-injection in a nude mice tumor model. Further, half-life of Cu-64 (12.7 h) was found to be compatible with the pharmacokinetics of PPF, thus providing adequate time for accumulation of ^64^Cu-PPF at tumor sites followed by PET imaging 168. Moreover, this concept was advanced to NPs, by direct inclusion of a radionuclide (Cu-64) into preformed organic, nontoxic, biodegradable porphysomes (porphyrin-lipid conjugates) forming ^64^Cu-porphysomes. As only a fraction of the porphysome is directly labeled with ^64^Cu, this preserves NIR fluorescence property of porphyrins. *In vivo* bimodal (PET/FL) imaging of ^64^Cu-porphysomes in tumor mice model, showed that ^64^Cu-porphysomes can clearly delineate tumor tissues 174,175. Further, Zheng et al., validated the *in vivo* sensitivity and selectivity of ^64^Cu-porphysomes in clinically relevant orthotopic prostate and bony metastatic cancer models by dual-mode PET/FLI. The study demonstrated ^64^Cu-porphysomes clearly delineated hypoxic orthotopic tumors on both the macro- and the microscopic scales and detected small bony metastases with high sensitivity 174. Later, the same group reported another strategy, where porphyrin (pyropheophorbide)-phospholipid (PoP)-coated UCNP was utilized for seamless post labelling with ^64^Cu for PET imaging. This ^64^Cu labeled PoP-UCNP demonstrated *in vivo* lymphatics imaging using 6 different imaging modalities via FLI, UCLI, PET, CT, Cerenkov luminescence, and PA tomography 20. The concept of seamless ^64^Cu labeling of porphyrins was expanded by developing a soluble Meso-tetra(4-carboxyphenyl) porphyrin (mTCPP)-PEG polymeric mesh as whole-body PET or multimodal imaging probe for renal function monitoring. PET and FLI imaging showed rapid clearance of mTCPP-PEG via renal excretion in healthy mice but not in mice with acute renal failure 176. Similarly, another surfactant-stripped micelle of pheophytin-a, was developed as a multimodal imaging agent. These Pheo ss-InFroMs were then seamlessly labeled with ^64^Cu for whole body gut imaging along with FLI and PAI 177.

Li et al., reported a robust, smart and highly versatile “all-in-one” porphyrin (pyropheophorbide)/cholic acid nanoconstruct (nanoporphyrin) which integrated several imaging (NIR FLI, PET, MRI,) and therapeutic (PTT, PDT) functions in a single entity. These nanoporphyrins were seamlessly labeled with ^64^Cu. PET imaging in ovarian cancer xenograft mice model revealed highest accumulation of nanoporhyrins in tumors at 24 h post injections with low background in the rest of the body. Further to gain accurate diagnosis this approach was extended by developing a dual-modality PET-MRI nanoprobe by incorporating both ^64^Cu(II) and Gd(III) into nanoporphyrins. Interestingly, combined MRI-PET imaging indicated inhomogeneous uptake and distribution of nanoporphyrins in the tumor 178. Feng et al., prepared a multipurpose liposome by encapsulating hydrophilic banoxantrone (AQ4N) into its aqueous cavity and hydrophobic hexadecylamine conjugated Ce6 in the hydrophobic bilayer, forming AQ4N-hCe6-liposome. These liposomes demonstrated improved therapeutic outcomes in tumor-bearing mice via sequential PDT and hypoxia-activated chemotherapy. Chelation of ^64^Cu with Ce6 formed PET probe AQ4N-64Cu-hCe6-liposome allowing an effective PET imaging together with FLI and PAI which indicated selective accumulation of liposomes in tumors 83.

Although ^64^Cu isotope has been extensively used for the synthesis of promising tumor-avid PS-based PET imaging agents. However, other isotopes have also been explored for the same. PSs have also been used for most common clinical PET radioisotope F-18 labeling. Li and colleagues used BF2 unit of BODIPY dyes as ^18^F- radiofluorination site. They utilized the ^18^F-PET/FL imaging modalities to study the biodistribution of the complex in *in vivo* tumor model which showed its maximum accumulation in the liver and kidneys 179. However, the short half-life of ^18^F limits its utilization for PS labeling and thus, alternatively, I-124 offers to a better candidate for PS labeling due to its long half-life of 4.2 days. MicroPET imaging revealed selective accumulation of Iodo-PK 11195 conjugated 3-[10-hexyloxyethyl]-3-devinyl pyropheophorbide-a HPPH in Translocator protein (TSPO) expressing tumor in mice model showing improved *in vivo* PDT efficacy 180. Pandey and colleagues also reported other ^124^I labeled purpurinimide and ^124^I- labeled Methyl Pyropheophorbide-a 146,181. Other than I-124, ^62^Zn (half-life 9.26 h) labeled glycoconjugated 5,10,15,20-tetrakis(pentafluorophenyl)porphyrin also proved its cancer-selective accumulation property through *in vivo* PET imaging, with tumor to normal tissue uptake ratio of four 182.

Similar to PET, SPECT is another radionuclide-based imaging modality that uses gamma-emitting radioisotope tracers like Technetium-99m, Iodine-123, Iodine-131, Indium-111. Gamma-rays (photons) emitted from SPECT radiotracers are then detected by collimated radiation detectors to generate images. Although both PET and SPECT have their own advantages and disadvantages, as compared to SPECT, PET provides better contrast and spatial resolution. Few studies have been carried out on utilization of PSs for chelation and imaging of SPECT tracers. Some of the examples includes Rhenium-188 (^188^Re)-ICG micelles, Gallium-67 (^67^Ga)-tetra phenyl porphyrin, Indium-111 (^111^In)- Meso tetrakis (4-hydroxyphenyl) porphyrin, Technetium-99m (^99m^Tc)-porphysomes, which have shown promising results for SPECT imaging for biodistribution and tumor uptake studies in mice model 183-186. Furthermore, in a clinical study, In-111-Photofrin-II was investigated for its uptake and distribution in 20 patients with intracranial neoplasms, using SPECT. The results showed variations in kinetics of complex uptake according to the tumor histology, the patient's use of steroids, and even among patients with similar types of tumor histology, which allowed personalized PDT planning 187.

Nuclear imaging modalities have the advantages of providing quantitative information at biochemical and molecular level with an excellent nanomolar to picomolar imaging sensitivity without deep tissue penetration limitations. However, as imaging modality, both PET and SPECT suffers from safety issue due to use of radionuclides, being costly with slow image acquisition times, and cannot be used for long-term studies due to radiolabel decay. Furthermore, clinicians always prefer to combine nuclear imaging with other complementary imaging techniques such as MRI and/or CT due to the lack of good spatial resolution and anatomical information in PET/SPECT imaging 161. Designing of PS-radioisotope conjugates or NPs requires careful consideration of the type of radionuclide and radiolabeling strategy, high *in vivo* stability and potential long-term toxicity. Another importance of employing radiolabelled (β-emitters) PS conjugates or NPs, is in Cherenkov Radiation Energy Transfer for exciting PS deep in the tissue without the application of an external light source as self-exciting “auto-PDT” 188. Radiolabeled PSs are useful for localizing the biodistribution of PS mainly in the tumor mass and for PS uptake studies; however, they cannot be used for visualization of post-PDT effects due to the loss of PS selectivity and uptake by tumor tissue. Consequently, different non-specific radiotracers are of interest to monitor and predict post-PDT response earlier than morphological imaging techniques like MRI and CT. Some of which includes: glucose metabolism with ^18^F-FDG for studying tumor vasculature damage and direct cell death; tumor proliferation with ^18^F-fluorodeoxythymidine; apoptosis with ^64^Cu-DOTA-biotin-Sav and ^99m^Tc-AnnexinV; hypoxia with ^123^I-iodoazomycin arabinoside and tumor vascular damage with 99mTc-hexakis-2-methoxyisobutyl isonitrile or 99mTc-hexamethylpropyleneamine oxime etc 164.

### Ultrasound Imaging combined Photodynamic Therapy

US imaging is one of the well-established, convenient and economical imaging techniques among all standard imaging modalities, due to its non-invasive real-time imaging nature, safety, non-usage of ionizing radiation, broad diagnostic applicability and easy handling. US imaging makes use of sound waves to produce an image of the tissue, based upon the differential interaction of sound waves with living tissue to provide quantitative structural and functional information of target organ and the velocity of moving tissue, primarily blood flow which can be easily visualized by US Doppler-effect mode. Furthermore, it is an established clinical imaging technique for identifying tumors and assessing disease progression via monitoring changes in tumor volume, lesion boundaries as cancer tissues have different echogenicity in comparison to healthy surrounding tissues. Thus, US imaging allows monitoring of structural, morphological and vasculature changes of tumor post-PDT and also provides information on apoptotic cell death 11,189.

Although conventional US imaging has better sensitivity to distinguish between normal and malignant tissues, its specificity is weak. In this regard, the use of US contrast agents provides improved sensitivity and high resolution of US imaging. Conventional US contrast agents are fluorinated gas-filled MBs stabilized by an outer shell of proteins, lipids, and polymers which are actively being used for Contrast Enhanced Ultrasound Imaging (CEUS). Cornelis et al., demonstrated the use of lyophilized MBs for CEUS imaging of tumor necrosis following WST11 (Tookad)‐based PDT‐inducing Vascular Targeted Photodynamic Therapy (VTP) in tumoral mice model. Although this study does not involve investigation of tumor growth kinetics or survival benefits based on CEUS of VTP of tumors, while the CEUS images showed good correlation with histology images of necrotic regions in tumors 190. Additionally, certain preclinical studies have investigated the use of MBs for molecular imaging, US-triggered drug and gene delivery. In this regard, Huynh et al., make use of MBs as PS delivery agents, wherein the “porshe microbubbles”, formed from a porphyrin-lipid shell encapsulating a fluorinated gas, for their *in vivo* US imaging of tumor mass in mice xenograft model 79,80. However, these MBs have a short half-life in circulation as they are easily destroyed by diffusing away from the gas at body temperature. Moreover, owing to their micron size, these MBs are not able to extravasate and accumulate in tumor mass or penetrate the deep tissue layers. Thus, this necessitates the need for developing effective US contrast agents with a long half-life, enhanced stability and tumor selectivity for tumor tissues diagnostics 191.

**Figure 8:**
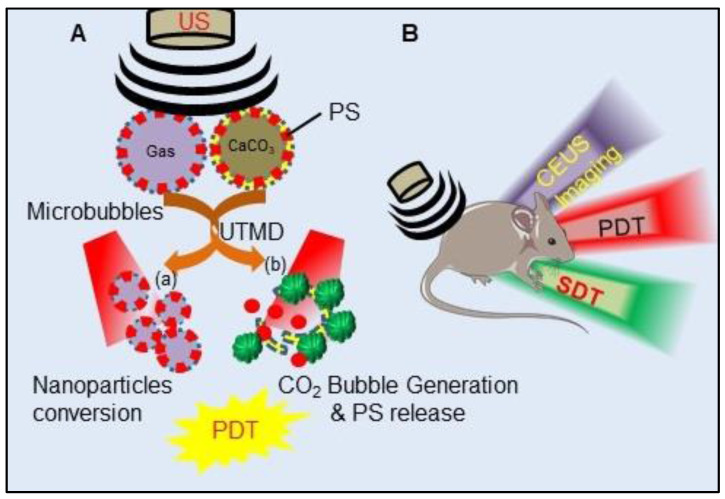
** Schematic illustration of microbubble mediated ultrasound-assisted imaging and guided Photodynamic Therapy (PDT): A.** Ultrasound (US) targeted microbubble destruction (UTMD) followed by (a) induced transformation of microbubbles to nanoparticles, (b) CO_2_ generation and photosensitizer (PS) release, resulting in tumoral uptake and *in vivo* PDT; **B.** Illustration of US induced Contrast Enhanced Ultrasound (CEUS) imaging, PDT and Sonodynamic Therapy (SDT).

Huynh et al., advanced the concept of porshe MBs to low-frequency US induced conversion of MBs to NPs. They synthesized perfluoropropane gas encapsulated bacteriochlorophyll-lipid shell MBs for trimodality contrast agents for US, PA and FL imaging, where the encapsulated gas provides US imaging contrast and the bacteriochlorophyll PS in the shell confers PA and fluorescent properties. Upon exposure to US, the MBs (1-10 μm) disintegrates and forms smaller NPs (5-500 nm) that retain the encapsulated payload of PSs and possesses the same optical properties as the original MBs. They further validated this concept in xenograft tumor-bearing mice model and suggested their PDT application 192. Later on, You et al., exploited the concept of US conversion of MBs to NPs and demonstrated that porphyrin-grafted lipid (PGL)-MB allowed favourable CEUS imaging outcome. Transformation of PGL-MBs into PGL-NPs upon US exposure, demonstrated exceptional accumulation ability at the tumor site via sonoporation effect. Thus, PS loaded MBs act as a good candidate for assisting targeted PDT in cancer theranostics, due to their enhanced US contrast to better localize tumor lesions, and targeted delivery of PS by virtue of ultrasound targeted microbubble destruction (UTMD) 193. The importance of nanobubbles (NBs) in CEUS imaging was further investigated by Huang and colleagues where they investigated NBs encapsulated mesoporous silica-coated gold nanorod UCNPs along with merocyanine 540 as PS (MC540-AuNR@UCNP@NB). This hybrid nanosytem allowed enhanced US imaging along with AuNR@UCNP mediated excitation of merocyanine 540 to improve NIR PDT 94. Park et al., reported Ce6-loaded pH-responsive bubble-generating CaCO_3_-mineralized NPs (Ce6-BMNs) having the potential for US imaging-guided PDT of tumors. Ce6-BMNs exhibited significant stability under physiological conditions and effectively inhibited Ce6 release at physiological pH (7.4). At tumoral acidic pH (6.4), dissolution of the CaCO_3_ mineral core allows CO_2_ bubble generation which resonates under applied US field and simultaneously triggers the release of Ce6 due to UTMD. This allows for greatly enhanced US signal-guided PDT for cancer theranostics 191. Figure 8 represents the schematic principle for the application of MBs mediated US-assisted imaging and guided PDT.

Similarly, some other PS-embedded CaCO_3_ NPs have been investigated for US imaging-guided PDT 194,195. Huang and colleagues reported the synthesis of dendritic mesoporous organosilica NPs encapsulating ICG and catalase (ICG-CAT@MONs) to overcome the tumor hypoxia induced PDT limitation and PA/US-guided tumor ablation. Upon light excitation, encapsulated ICG results in simultaneous PAI and PDT of *in vivo* tumor tissues, while the catalase molecules catalyze the decomposition of endogenous H_2_O_2_ into O_2_ bubbles to intensify US imaging signal and enhance PDT efficacy simultaneously. Thus, the results showed excitation of ICG-CAT@MONs causes enhanced tumor destruction due to the sustainable O_2_ generation, eventually promoting PDT and holds promise in multimodal PA/US image-guided PDT 196. In another exciting discovery, Sun and collaborators reported the synthesis of multifunctional PGL MBs loaded with HIF-1α siRNA (siHIF@CpMB) for PDT combined with gene therapy in triple-negative breast cancer therapy. US imaging allowed real-time monitoring of siHIF@CpMB distribution. Furthermore, UTMD of siHIF@CpMB efficiently converts them into NPs promoting the accumulation of porphyrin and siRNA at the tumor site via the cavitation effect. HIF-1α siRNA downregulates HIF1α expression in the hypoxic tumor region, ultimately relieving hypoxia-induced ROS generation during PDT and thus improves the overall therapeutic outcome. In a similar study, PGL MBs were shown to achieve US-controlled PS accumulation in tumors and enhanced US-assisted PDT efficacy in *in vivo* prostate cancer mice model 197.

Presently, the US technique is widely applied for imaging and diagnostics in clinical setups, owing to its safety, non-invasiveness, good tissue penetration ability, low operation, and instrumental costs. Further, targeted PS based CEUS is an emerging imaging strategy for evaluating biological processes at the molecular level, also known as the molecular US imaging, which is based on sophisticated PS - US contrast agent designing as discussed in this section. However, US imaging suffers from low resolution, and usually fails to accurately determine lesion size, boundaries, and fine details of the anatomy of deep tissue which leads to operator variability. Further technical advancements like the introduction of better light delivery systems, US transducers with better sensitivity, faster data acquisition, advanced computed 3D reconstruction and better image processing algorithms can overcome the inherent limitations and will establish the utility of US imaging in PDT dosimetry and treatment monitoring 11. Many commonly used PSs such as Hp, ALA, Ce6, Pheophorbide a, ZnPc etc., have shown to act as sonosensitizers. Activation of sonosensitizers by US exerts anticancer therapeutic effects through sonodynamic therapy (SDT) by generating ROS. Consequently, the combination of PDT and SDT delivers improved therapeutic effects from surface to the significant tissue depth, due to deep tissue targeting by focused US energy compared to visible light penetration 198.

### X-ray Computed Tomography combined Photodynamic Therapy

CT imaging is an age-old computerized X-ray based, cost-effective imaging modality in clinics that uses the combination of a narrow beam of X-rays to generate cross-sectional images or “slices” of the body, known as tomographic images. Numerous successive slices are then digitally “stacked” together to form a 3D image of the body or body organs that allows localizing possible tumors. As compared to conventional X-ray images, CT generates a cross-sectional image with 3D anatomic details for both soft and hard tissues. In clinics, X-ray attenuating contrast media containing heavy elements (most commonly Iodine or Barium) are used to generate CT images of soft tissues with high contrast to the surrounding tissues 199,200. In this regard many heavy atoms based nanoformulations have been utilized for CT imaging combined PDT. For example, Yin and colleagues utilized gold as an X-ray attenuation element to develop a meso-tetrakis(4-carboxyl)-21H,23H-porphine (TCPP) covalent organic polymer (COP-8) and β-cyclodextrin (β-CD) functional AuNPs, COP@Au@TCPP nanocomposites for gold-based CT-image-guided PDT. Moreover, decomposition of H_2_O_2_ into O_2_ catalyzed by CD-AuNPs in tumor cells further assisted in overcoming the limitation of hypoxia-induced PDT efficacy of TCPP 200. In another study, a biocompatible PEGylated nanoliposomes co-encapsulating clinically approved Iodinated CTIA iodixanol (Visipaque®) and a PS TPPS4 demonstrated enhanced PDT guided by CT imaging 201. Similarly, many other nanoformulations combing heavy element and PS have been reported as a promising agent for CT imaging combined with PDT 86,202-208.

X-ray CT is one of the most extensively used cost-effective clinical imaging techniques which allow deep tissue and/or whole-body imaging with high spatial resolution and 3D visualization with fast image acquisition and processing time. However, as compared to MRI, CT suffers from safety issues due to the use of high-energy ionizing radiations and also less efficient in contrasting soft tissues. Combination of CT with other imaging techniques like highly sensitive OI and molecular imaging like MRI and nuclear imaging techniques can exhibit great advantages in cancer theranostics. However, studies of CT using PS-based CT contrast agents have been restricted to *in vivo* small animals using microCT. Therefore, improvement in detectors and spatiotemporal CT scanners hold promise for deployment of CT in clinics as multimodal imaging advancements.

## Tumor Microenvironment Responsive Activatable Photosensitizers: For Selective Photo-Theranostics

Although image-guided laser irradiation and targeted PS provides preferential tumor targeting, they still cannot completely overcome the side effects of non-targeted tissue photodamage. Therefore, an advanced strategy for developing aPS with higher selectivity and efficiencies as smart phototheranostic agents has gained current research interest 4,27. As compared to traditional PSs, activatable phototheranostic PSs are strategically designed to respond to cancer-specific environmental stimuli which allow both molecular imaging and phototoxic effects at the target tumor site. More importantly, even after light exposure, aPS remains passive or unexcited in the absence of tumor-associated stimuli. This strategy allows higher tumor-selective accumulation and efficacy with minimal cytotoxicity to surrounding healthy cells and further offers higher imaging sensitivity as tumor molecular diagnostics demarcates target cancerous tissues from healthy tissues 33. Designing and developing efficient aPSs needs to take into consideration (i) introducing proper stimuli-responsive linkers or groups; (ii) properties like high PS activation efficiency including both ^1^O_2_ quenching in its deactivated state and reactivation; to impart high therapeutic efficacy of PDT with minimum side effects. Various natural and biodegradable polymers e.g. polylactide (PLA), poly(ε-caprolactone) (PCL), poly(lactic-co-glycolic acid) (PLGA), pluronic, polyphosphoester, hyaluronic acid (HA), dextran, and chondroitin sulfate have been utilized as stimuli-responsive polymeric nanovehicles for delivery of various PSs. PSs are usually introduced into these polymeric nanoformulations either by chemical conjugation with the polymers or physically being encapsulated into nanosystems 67,209. Advantages of stimuli-responsive nanoformulation involve higher stability and reduced premature activation of PS, due to its self-quenching effect, which all together minimizes the side effects while in circulation and only are activated in targeted sites to induce effective PDT outcomes. Designing strategies of various novel stimuli-responsive PS based nanocarriers in response to various intracellular and tumor microenvironment-based stimuli such as pH, antioxidants, specific enzymes, ATP, ROS, redox-potential and hypoxia have already been reviewed 4,6,27,33,34,210-212. The following section presents some representative tumor microenvironment-responsive aPSs inducing targeted phototoxicity combined with various imaging modalities (Table 4).

In comparison with conventional “always-on” PSs, tumor microenvironment responsive “turn-on” PS nanoformulations offer the advantages of improving the sensitivity and resolution of imaging modalities. aPSs usually amplify imaging signals at the target site together with reducing the background signals and/or autofluorescence (for FLI), which consequently increases the SNR and reduces the limit of detection.

## Conclusions and Future perspective

Since the first approval of PDT in 1995 as an esophageal cancer treatment modality, due to its feasibility and effectiveness, it has made conspicuous progress clinically to establish itself as a treatment strategy for other cancers as well. Although PDT has proved its tremendous potential for being an efficacious treatment strategy against cancer, its application is still restricted as an adjunct therapy and palliative care for cancer patients 11. One of the main setbacks in PDT or other cancer therapies involve lack of proper dosimetric strategies in evaluating actual drug dose at the tumor site for envisaging therapeutic response and planning of subsequent therapies. Molecular imaging modalities like conventional OI, MRI, PET, US and CT are able to provide detailed structural, functional and molecular information about pre- and post-therapeutic conditions of the tumor. However, they cannot provide real-time monitoring because of the use of different agents for imaging and therapy. In this regard due to its favorable and fascinating chemistry, PSs can be easily modified into theranostic agents for application in almost all available imaging techniques or multimodality imaging combined with PDT 231,232. In this review, we have discussed molecular design rationales for PSs that have been pursued for the development of multifunctional theranostic agents. Nanotechnology has further widened the scope of this research area as it allows to explore ''two-in-one'' or ''multiple-in-one'' imaging parameters in a single nanoformulation along with tumor-targeting properties. Application of smart aPS summarized in this review can simultaneously activate their imaging signals and phototoxic effects in response to cancer-specific stimuli such as pH, ROS, cancer-associated enzymes, proteins etc. Compared to always-on theranostic agents these aPSs have the advantages of (a) higher diagnostic specificity and sensitivity, (b) improved and selective treatment efficacy, and (c) minimum systematic side-effects.

Although the research of developing PS-based theranostic agents with multimodal imaging abilities has recently gained momentum, they are still under the growth stage. Despite the progress in strategic designing of novel PS-based theranostic agents, currently available non-activatable and activatable formulations are majorly based on the inherent FLI-guided PDT. Although other imaging techniques offer more effective and accurate diagnostic information, due to the limited choice of materials/metals, they have been less explored. As the designing and development of such multifunctional agents are mainly based on nanomaterial formulations and nonbiological elements, few serious issues need to be addressed before pushing such agents into clinical trials. Dose, pharmacokinetics and potential toxicity of such formulations need to be further investigated for the purpose of clinical translation. Further, the *in vivo* metabolism of nanomaterials should be studied in detail as their clearance pathway is through the reticuloendothelial system. Especially non-degradable nanomaterials require weeks for clearance from the body and accumulates in high concentration in the reticuloendothelial organs increasing the risk of damage to the human body and organs. Hence, clearance optimization of such nanomaterial-based agents needs to designed in such a manner as to reduce their dimensions (∼5 nm) by enhancing their *in vivo* biodegradability for renal clearance 33,233. Other than strategic designing, and *in vivo* toxicity and effectiveness assessment of phototheranostic agents, several technical obstacles need to be addressed for each imaging modalities. As already discussed, all the imaging techniques suffer from their intrinsic limitations, however, technical advancement at the image acquisition and processing level such as improvement in sensitivity of detectors and equipments, image processing and 3D reconstruction algorithms and faster data acquisition can assure better imaging outcomes for effective PDT.

**Figure 9:**
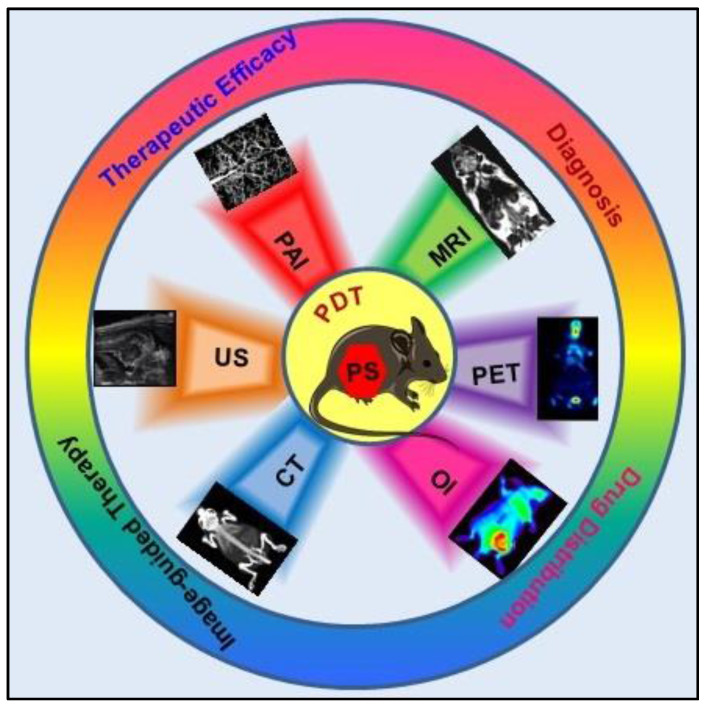
** Theranostic Photosensitizers:** Applications of theranostic photosensitizers (PS) in various imaging modalities: Magnetic Resonance Imaging (MRI), Positron Emission Tomography (PET), Optical Imaging (OI), X-ray computerized tomography (CT), Ultrasound Imaging (US), Photoacoustic Imaging (PAI).

In summary, as represented in Figure 9, PS based multifunctional theranostic agents hold great potential to make personalized and integrated therapy feasible. Further, integrating different clinically available MRI, CT, PET, US with more advanced imaging modalities like optical, PA, SERS increases imaging penetration depth, diagnosis accuracy, and sensitivity for efficacious image-guided PDT to envision the era of personalized medicine.

## Figures and Tables

**Table 1:** Characteristics of clinical molecular imaging modalities in oncology.

	PET	SPECT	CT	MRI	US	OPTICAL
**Signal Used**	High-energy γ-ray	Low-energy γ-ray	X-rays	Radio waves	High-frequency sound waves	Visible light or near-infrared
**Contrast agents/Tracers**	β+ emitting Radioisotope	γ- emitting Radioisotope	Krypton, Xenon, Barium and iodinated molecules	Gadolinium chelates/ superparamagnetic agents (SPIONs)	Microbubbles	Fluorescent probes/ dyes
**Sensitivity^a^ (mol/L)**	10^-11^-10^-12^	10^-10^-10^-11^	Not well characterized	10^-3^-10^-5^	Not well characterized	10^-9^-10^-12^
**Spatial Resolution^b^**	1-2 mm	1-2 mm	50-200 μm	25-100 μm	50-500 μm	2-3 mm
**Temporal Resolution^c^**	10 seconds to minutes	Mins	Mins	Mins-Hrs	Sec-Min	Sec-Min
**Depth of Penetration**	No limit	No limit	No limit	No limit	mm-cm	< 1 cm
**Advantages**	High sensitivity, can be used for whole body imaging		High resolution, can be used for whole body imaging, fast acquisition time	High spatial resolution, no ionizing radiation, high soft tissue contrast	Fast acquisition time, real-time imaging, no ionizing radiation, cost-effective	Fast acquisition time, no ionizing radiation, real-time imaging, high sensitivity, cost-effective
**Disadvantages**	Ionizing radiations, low resolution, expensive, long acquisition time		Ionizing radiations, low sensitivity, poor soft tissue demarcation	Poor sensitivity, long acquisition time, expensive	Poor contrast, low resolution	Low Resolution

^a^Sensitivity is the ability of imaging technique to detect or identify the presence of a molecular probe when it is truly present, relative to its background. ^b^Spatial resolution is a measure of the accuracy or detail of image. It is mainly based on its detection ability to distinguish two adjacent structures as separate entities. ^c^Temporal resolution is the frequency at which the images are be recorded or captured. It is also represented as single acquisition time. PET: Positron Emission Tomography; SPECT: Single-Photon Emission Computerized Tomography; CT: Computed tomography; MRI: Magnetic Resonance Imaging; US: Ultrasound

**Table 2:** Summary of clinically relevant oncology informations provided by different imaging modalities to guide, monitor and evaluate Photodynamic Therapy responses in preclinical and clinical settings.

Imaging Modality	Pre and Post treatment Information
**Endoscope-coupled FL, US, OCT**	Tumor localization in hollow tube organs
**CT**	Tumor localization Necrosis and surviving tumor volume
**MRI**	Tumor volume Necrosis and histopathological analysis Vascular perfusion and permeability
**PET**	Tumor volume and localization Tumor hypoxia Necrosis and surviving tumor volume
**OCT**	Microscopic resolution of Tumor volume and margin delineation of superficial tumors
**Laser Doppler imaging and Angiography**	Vascular perfusion and blood flow velocity
**OI**	FL based PS uptake and photobleaching mediated treatment response Singlet oxygen luminescence (SOL)
**US**	Tumor volume and localization Necrotic and apoptotic tumor fraction Vascular density, perfusion and blood flow velocity Image-guided fiber placement
**PAI**	Vascular structure and density PS uptake and distribution PS photobleaching rate Dynamics of blood oxygen saturation and partial pressure of oxygen

PET: Positron Emission Tomography; SPECT: Single-Photon Emission Computerized Tomography; CT: Computed tomography; MRI: Magnetic Resonance Imaging; US: Ultrasound Imaging; FL: Fluorescence Imaging, OI: Optical Imaging; OCT: Optical Coherence Tomography; PAI: Photoacoustic Imaging; PS: Photosensitizer.

**Table 3:** List of photosensitizers approved or in clinical trials for Photodynamic Therapy and diagnosis in oncology.

Class	Examples	λ_max_	Clinical Approval
**A. Tetrapyrrole based**			
***(i) First generation***			
Porphyrin 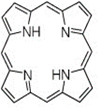	(a) Porfimer sodium (photofrin) 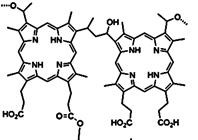	630 nm	Approved-Bladder cancer, Endobronchial cancer, Esophageal cancer, Lung cancer, Barrett's esophagus, cervical cancer In clinical trial- Brain cancer diagnosis
*(ii) Second generation*			
Porphyrin precursor	(a) 5-Aminolevulinic acid (Levulan) 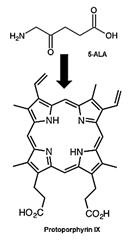	635 nm	Approved- Non-melanoma skin cancers, Basal cell carcinoma, Squamous cell carcinoma In clinical trial- Brain cancer diagnosis and guided resection
	(b) Hexaminolevulinate hydrochloride (Hexvix®) 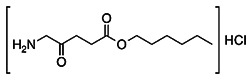	635 nm	Bladder cancer diagnosis
Chlorin 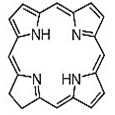	(a) 5,10,15,20-Tetrakis(3-hydroxyphenyl) chlorin/ Temoporfin (Foscan) 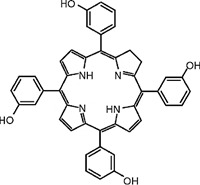	652 nm	Approved- Head and neck, Prostate and Pancreatic cancers
	(b) Mono-L-aspartyl chlorin e6 / Talaporfin (Laserphyrin) 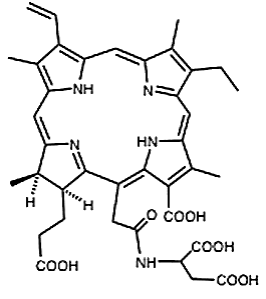	664 nm	Approved- Lung cancer, Malignant gliomas
Bacteriopheophorbide 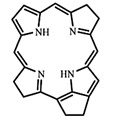	(a) Palladium-Bacteriopheophorbide (WST09)/ Padoporfin (Tookad) 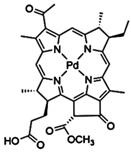	763 nm	Approved- Prostate cancer
			
	(b) Bacteriopheophorbide (WST11)/ padeliporfin (Stakel) 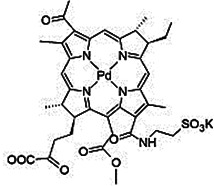	~ 750 nm	In clinical trials- Prostate cancer
Purpurin	Tin ethyl etiopurpurin/ Rostaporfin (Purlytin) 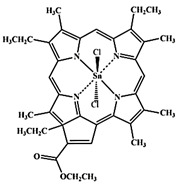	664 nm	In clinical trials- Basal cell cancer, Kaposi's sarcoma, Prostate cancer, Breast adenocarcinoma
Pheophorbide 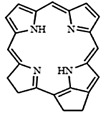	2-(1-Hexyloxyethyl)-2-devinyl pyropheophorbide-a / PhotoChlor 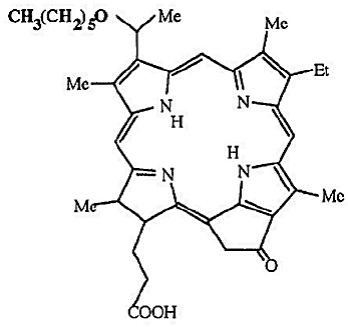	665 nm	In clinical trials- Basal cell carcinoma, Esophagus, Skin, Mouth and Throat cancers, Cervical intraepithelial neoplasia
Texaphyrin 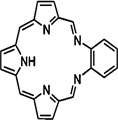	Motexafin lutetium (Antrin/ Lutrin) 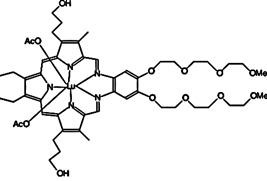	732 nm	In clinical trials- Prostate, Breast, Cervical, Brain, Skin and Superficial cancers
Porphyrin related-Phthalocyanine (Pc) 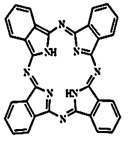	(a) Aluminum phthalocyanine tetrasulfonate chloride (Photosens) 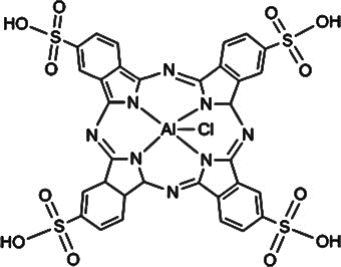	676 nm	In clinical trials- Stomach, Skin, Lip, Oral, and Breast cancers
	(b) Zinc pthalocyanine 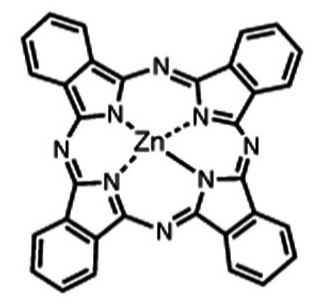	676 nm	In clinical trials- Skin cancer, Squamous cell carcinoma
	(c) Silicon Phthalocyanine 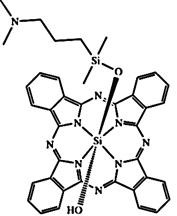	675 nm	In clinical trials- Skin cancer
	B. Non-Porphyrin based		
Anthraquinone 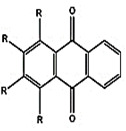	Hypericin 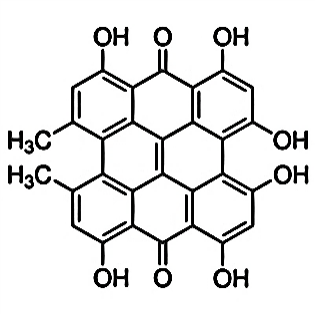	600 nm	In clinical trials- Cutaneous T-cell Lymphoma
Cyanine	Indocyanine green (IR125) 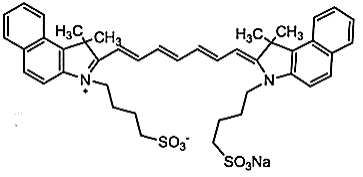	695-780 nm (Concentration dependent)	In clinical trials- Imaging-guided detection and PDT

**Table 4:** Representative theranostic activatable photosensitizers as conjugates and nanoparticles for simultaneous imaging and Photodynamic Therapy.

Activating factor	PS and Nanoformulation	Targeting moiety	Imaging technique	Reference
Low pH	aza-BODIPY (NEt_2_Br_2_BDP) Cyclic arginine-glycine-aspartate motif (cRGD)-functionalized nanomicelle	cRGD for αvβ3 integrin-rich tumor cells.	NIR	213
	Chlorin e6 (CaCO_3_-mineralized NPs)	--	US	191
	Methylene Blue (CaCO_3_ nanorods)	--	US	195
	Pheophobide-a (FA-BSA-c-PheoA)	Folate	NIR	214
	Zn(II) phthalocyanine (Layered double hydroxide-PcS supramolecular nanohybrid)	--	FLI	215
	Sinoporphyrin sodium (Manganese-doped calcium phosphate mineralized glucose oxidase nanoparticles)	--	FLI and MRI	65
	Indocyanine green (Mesoporous silica-coated gold nanorods)	--	FLI and PAI	216
Hyaluronidase	Indocyanine green (MnO_2_ modified hyaluronic acid NPs)	Hyaluronic acid for CD44 receptor binding	FL and PAI	217
	Chlorin e6 (HA-ADH-Ce6 NPs)	Hyaluronic acid for CD44 receptor binding	NIR and PAI	218
Reducing TME	Chlorin e6 (Ce6-heparin- alpha-tocopherol succinate)	--	NIR	219
	Chlorin e6 (Ce6- dextran conjugates nanoformulations)	--	NIR	220
	Chlorin e6 (Ce6-fucoidan conjugates nanogels)	Fucoidan for P-selectin surface protein	NIR	221
	Chlorin e6 (α-cyclodextrin (α-CD) and poly (ethylene glycol) -Ce6)	--	NIR	222
Low pH & reducing TME	Toludine blue (FeOOH modified NaLuF4:Yb,Er,Tm@NaLuF4)	--	UCL	95
	Chlorin e6 (CNT@MnO_2_-PEG@Ce6 carbon nano tubes)	Folate terminated aminated poly (ethylene glycol) (FA-PEG-NH2)	MRI	223
H_2_O_2_	Pro-photosensitizer (MBPB) converted to active methylene blue (BSA-MBPB nanoformulations)	--	NIR and PAI	224
Low pH and H_2_O_2_	Chlorin e6 (Ce6/MnOx@HMSNs-PEG)	--	MRI	225
	Methylene blue (SiO_2_-MB@MnO_2_)	--	MRI	226
Reducing TME and matrix metalloproteinase	Chlorin e6 (PEGylated Ce6-MMP2 NPs)	Matrix metalloproteinase 2	NIR	227
Biomolecules responsive				
Albumin	Zn(II) phthalocyanine (Self-assembled supramolecular nanovesicle)	__	FLI	228
Nucleic acid	Zn(II) phthalocyanine (Mitoxantrone and Zn(II) phthalocyanine supramolecular nanoassembly)	__	FLI	229
Biotin	Zn(II) phthalocyanine (Phthalocyanine - triethylene glycol self-assembled nanoformulations)	Biotin moiety	FLI and PAI	230

NIR: Near Infrared Imaging, MRI: Magnetic Resonance Imaging; PAI: Photoacoustic Imaging; UCL: Upconversion Luminescence Imaging; US: Ultrasound Imaging; FLI: Fluorescence Imaging; NPs: Nanoparticles; TME: Tumor microenvironment

## Abbreviations

^1^O_2_: Single Oxygen
AIE: Aggregation Induced Emission
5-ALA: 5-aminolevulinic acid
AlPcS4: Aluminum phthalocyanine tetrasulfonic Acid
aPS: Activatable photosensitizer
AgNPs: Silver Nanoparticles
AuNPs: Gold nanoparticles
BHQ: Black Hole Quenchers
Ce6: Chlorin e6
CEUS: Contrast Enhanced Ultrasound Imaging
CT: Computed Tomography
EPR: Enhanced Permeation and Retention
FA: Folic Acid
FDG: Fluorodeoxyglucose
FGS: Fluorescence-guided surgery
FL: Fluorescence
FLI: Fluorescence Imaging
GSH: Reduced Glutathione
GdDTPA: Gadolinium-diethylenetriaminepentacetate
Gd-DTTA: Gadolinium-(siloxylpropyl)diethylenetriamine tetraacetate
GdDOTA: Gadolinium-tetraazacyclododecane tetraacetic acid
ICG: Indocyanine green
ISC: Intersystem crossing
HA: Hyaluronic acid
Hp: Hematoporphyrin
HPPH: 3-devinyl-3-(1-hexyloxyethyl) pyropheophorbide-a
H_2_O_2_: Hydrogen peroxide
HSA: Human Serum Albumin
MB: Methylene blue
MBs: Microbubbles
MNPs: Magnetic Nanoparticles
MR: Magnetic Resonance
MRI: Magnetic Resonance Imaging
NBs: Nanobubbles
NIR: Near Infrared Radiation
NP: Nanoparticle
OCT: Optical Coherence Tomography
OI: Optical Imaging
PA: Photoacoustic
PAI: Photoacoustic imaging
PcS4: phthalocyanine tetrasulfonic acid
PDD: Photodynamic Diagnosis
PDT: Photodynamic therapy
PEG: Polyethylene Glycol
PGL: porphyrin-grafted lipid
PET: Positron Emission Tomography
PFD: Photosensitizer fluorescence detection
PpIX: Protoporphyrin IX
PS: Photosensitizers
PTT: Photothermal Therapy
RB: Rose Bengal
RIM: Restriction of intramolecular motions
ROS: Reactive Oxygen species
SERS: Surface-enhanced Raman scattering
SNR: Sound to Noise ratio
SPECT: Single-Photon Emission Computed Tomography
SPION: Superparamagnetic iron oxide nanoparticle
Sq: 2,4-bis[4-(N,N-dibenzylamino)-2,6-dihydroxyphenyl] squaraine
TB: Toludine Blue
TPP: Tetraphenylporphin
TPPS4: Meso-tetra-(4-sulfonatophenyl) porphyrin
Tz: Tetrazine
UCL: Upconversion luminescence
UCLI: Upconversion luminescence Imaging
UCNP: Upconversion nanoparticle
US: Ultrasound
UTMD: Ultrasound targeted microbubble destruction
ZnPc: Zinc Phthalocyanine
